# Traditional Uses, Botany, Phytochemistry, Pharmacology, Pharmacokinetics and Toxicology of *Xanthium strumarium* L.: A Review

**DOI:** 10.3390/molecules24020359

**Published:** 2019-01-19

**Authors:** Wenxiang Fan, Linhong Fan, Chengyi Peng, Qing Zhang, Li Wang, Lin Li, Jiaolong Wang, Dayong Zhang, Wei Peng, Chunjie Wu

**Affiliations:** 1 School of Pharmacy, Chengdu University of Traditional Chinese Medicine, Chengdu 611137, China; fwx13990706098@163.com (W.F.); fanlinhong1996@163.com (L.F.); CEandAnthony@163.com (C.P.); zq1995729@163.com (Q.Z.); liwang201812@163.com (L.W.); li54627627@163.com (L.L.); tcmwangjiaolong@163.com (J.W.); zdy@xinhehua.com (D.Z.); 2 Sichuan Neautus Traditional Chinese Herb Limited Company, Chengdu 611731, China

**Keywords:** *Xanthium strumarium* L., traditional usages, botany, phytochemistry, pharmacology, pharmacokinetics, toxicology

## Abstract

*Xanthium strumarium* L. (Asteraceae) is a common and well-known traditional Chinese herbal medicine usually named Cang-Er-Zi, and has been used for thousands of years in China. The purpose of this paper is to summarize the progress of modern research, and provide a systematic review on the traditional usages, botany, phytochemistry, pharmacology, pharmacokinetics, and toxicology of the *X. strumarium*. Moreover, an in-depth discussion of some valuable issues and possible development for future research on this plant is also given. *X. strumarium*, as a traditional herbal medicine, has been extensively applied to treat many diseases, such as rhinitis, nasal sinusitis, headache, gastric ulcer, urticaria, rheumatism bacterial, fungal infections and arthritis. Up to now, more than 170 chemical constituents have been isolated and identified from *X. strumarium*, including sesquiterpenoids, phenylpropenoids, lignanoids, coumarins, steroids, glycosides, flavonoids, thiazides, anthraquinones, naphthoquinones and other compounds. Modern research shows that the extracts and compounds from *X. strumarium* possess wide-ranging pharmacological effects, including anti- allergic rhinitis (AR) effects, anti-tumor effects, anti-inflammatory and analgesic effects, insecticide and antiparasitic effects, antioxidant effects, antibacterial and antifungal effects, antidiabetic effects, antilipidemic effects and antiviral effects. However, further research should focus on investigating bioactive compounds and demonstrate the mechanism of its detoxification, and more reasonable quality control standards for *X. strumarium* should also be established.

## 1. Introduction

Since 1963, the fruits of *Xanthium strumarium* L. have been listed in the Pharmacopoeia of the People’s Republic of China (CH.P), and currently over 60 formulas containing the fruits of *X. strumarium* have been applied for treating various diseases, including rhinitis, nasal sinusitis, headache, gastric ulcer, urticarial, rheumatism, bacterial and fungal infections, and arthritis [1,2,3]. So far, many studies have been devoted to the pharmacological and phytochemical studies of *X. strumarium*, and more than 170 chemical compounds have been isolated and identified from this plant, including sesquiterpene lactones, phenols, glycoside, alkaloids, fatty acid and others [4]. In addition, increasing evidence has indicated that *X. strumarium* possesses a wide spectrum of pharmacological activities including analgesic and anti-inflammatory, antioxidant, hypoglycemic, anti-cancer, antibacterial and antifungal, anti-trypanosomal, anti-tussive activities, and effects on nervous and digestive systems, as well as other effects [1]. Nowadays, the fruits of *X. strumarium* remains a common Traditional Chinese Medicine (TCM) listed in the CH.P, and atractyloside and chlorogenic acid are used as the quality indicator agents for evaluating quality of the fruits of *X. strumarium* [5].

In this paper, we systematically summarize the traditional uses, botany, phytochemistry, pharmacology, pharmacokinetics as well as the safety aspects of *X. strumarium*, hoping that it could propel the research forward for applying the medicinal values of this plant completely. Moreover, potential research directions and emphasis on *Xanthium strumarium* L. are discussed as well.

## 2. Traditional Usages

*X. strumarium* has a long history for utilization as a medicinal plant in China due to its extensive biological and pharmacological activities. In particular, the fruit is the predominant medicinal part of *X. strumarium*, and is one of the most common used herbal medicines to treat rhinitis and headache for thousands years [6]. Before clinical use, the fruits of *X. strumarium* are often processed by stir-baking to a yellowish color, which aims to reduce toxicity and enhance efficacy. The first record of the pharmacological effects of this plant can be traced back to ShenNong BenCaoJing, which is the earliest monograph of TCM during the Eastern Han dynasty. In this monograph, it was used for the treatment of anemofrigid headache and rheumatic arthralgia. Then, in Mingyi Bielu which is another known TCM monograph, X. strumarium was recorded as an effective herbal medicine with the function of curing gonyalgia. In Yaoxinglun, X. strumarium was described as an agent for treating hepatic heat and eye diseases. Subsequently, another famous monograph, Xinxiu Bencao, described X. strumarium with improving eyesight, antiepileptic and antirheumatic properties. Besides, X. strumarium was also listed in some other classical monographs of materia medica in China, such as Bencao Shiyi, Bencao Mengquan, Depei Bencao, Caomu Bianfang, Tianbao Bencao and others. 

Currently, the fruits of *X. strumarium* have become an important traditional Chinese medicine commonly used in clinic for the treatment of nasal diseases (including acute and chronic rhinitis, allergic rhinitis (AR), nasosinusitis, and nasal obstruction), itching diseases, and painful diseases. In order to meet clinical needs better, various forms of formulas are developed, such as pills, tablets, granules, oral liquid, powders and others (Table 1). Furthermore, in India, *X. strumarium*, commonly known as Chotagokhru or Chotadhatura, are usually used to cure leucoderma, poisonous bites of insects, epilepsy, and biliousness [7]. In addition, several North American Indian tribes and Zuni tribes apply this plant to relieve constipation, diarrhoea and vomiting [1]. Besides, *X. strumarium* is also reported as a folk herbal medicine in Bangladesh for the treatment of urinary disorder, ear infection, diabetic, and gastric disorder [8]. 

Apart from clinical application, its potential capacity as a biodiesel feedstock has been proven. *X. strumarium* has very strong environmental adaptability and thus has numerous wild resources. The seed has a high oil content (42.34%) which gives potential annual output of 100,000 tons just in China [9]. Furthermore, the research in Pakistan also found the prospects of non-edible seed oils for use as biodiesel to solve the serious energy crisis [10].

## 3. Botany

**Figure 1:**
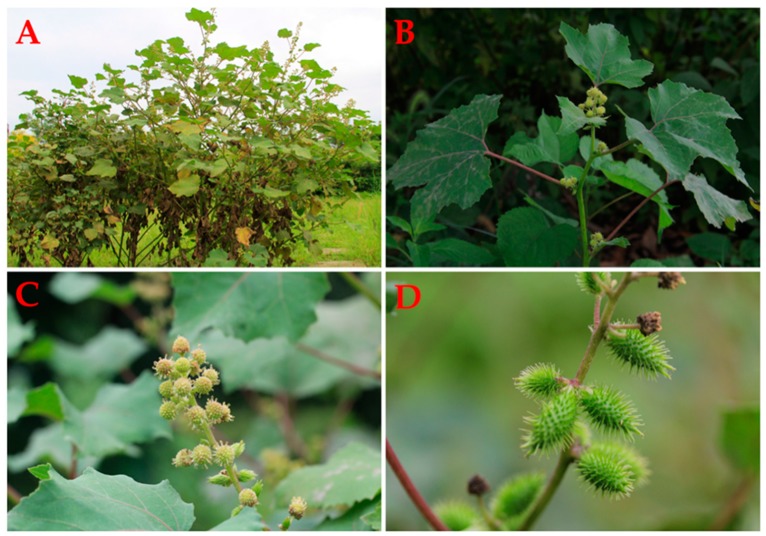
*Xanthium strumarium* L. A–D represent the whole plants (**A**), leaves (**B**), inflorescence (**C**) and fruits (**D**) of *X. strumarium* L.

*Xanthium*, belonging to the Asteraceae family, is a taxonomically complex genus, which includes more than 20 species in the world and three species and one varietas in China [8]. *Xanthium strumarium* L. (Figure 1) is an annual herb approximately 20–90 cm in height, its stems are erect, branched, often speckled with purple and have short white hairs scattered across the surface. Leaves are green, cauline, mostly alternate (proximal 2–6 sometimes opposite) with petiole, which are 5–20 cm long and 4–16 cm wide; the shape of blades are lanceolate, linear, ovate, orbicular-deltate, or suborbicular, and both surfaces are hirtellous or strigose, usually with gland-dotted, margin entire or toothed. The capitula are discoid, whose female (proximal) or functionally male (distal) are in racemiform to spiciform arrays or borne singly (in axils). The female capitula are elliptic, 2–5 mm in diameter; Male capitula are saucer-shaped, 3–5 mm in diameter. The achenes are black, fusiform, obovoid, enclosed in the hardened involucre, with two hooked beaks and hooked bristles [11,12]. 

This plant is widely distributed all over the world, including Russia, Iran, India, North Korea and Japan. It is native to China and widely distributed in the area of Northeast China, Southwest China, North China, East China and South China. It often grows in plains, hills, mountains and wilderness roadsides. The flowering time ranges from July to August, and fruiting stage lasts from September to October in China [1].

## 4. Phytochemistry

**Figure 2:**
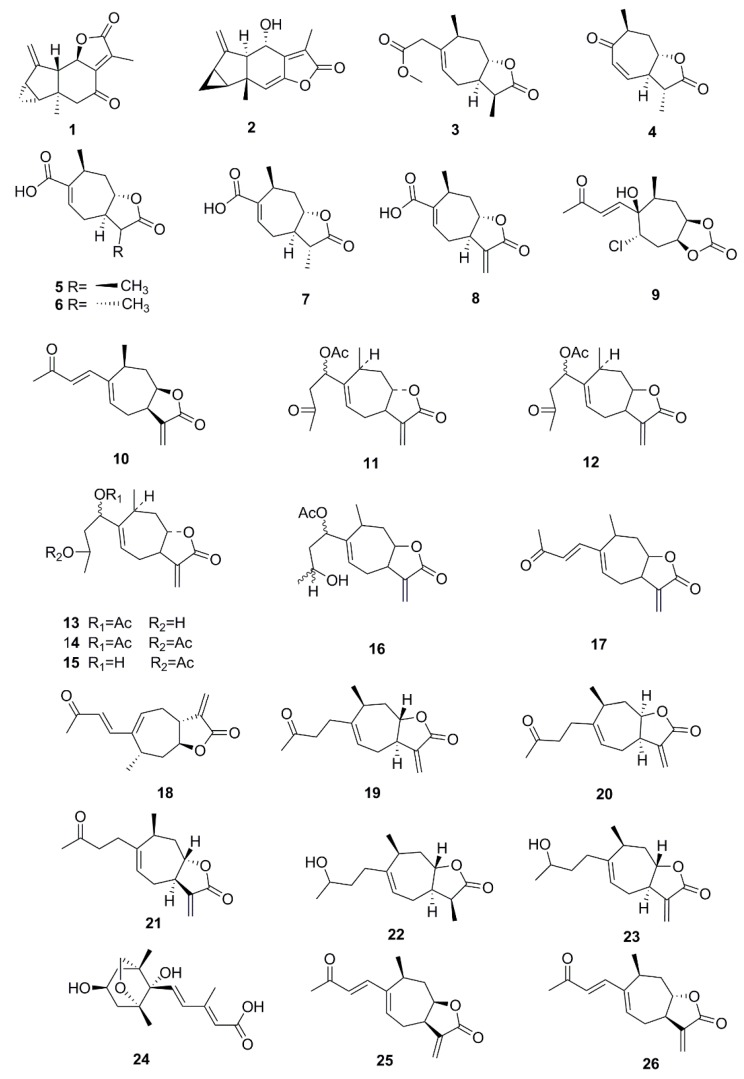

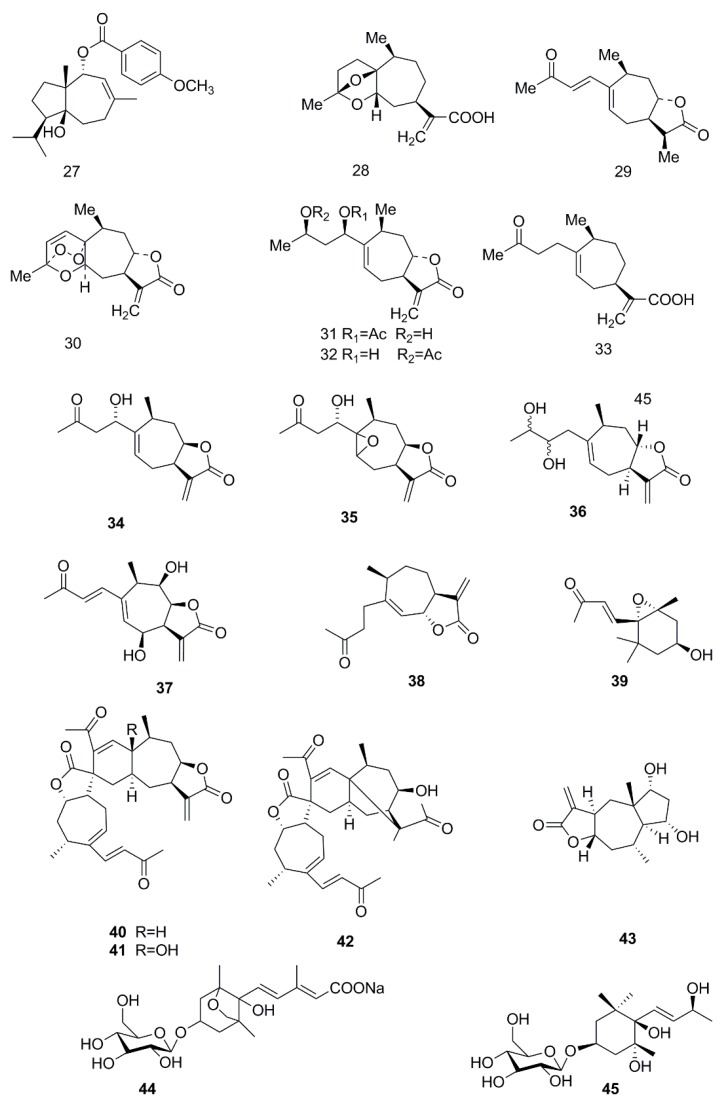
Chemical structures of the sesquiterpenoids in *X. strumarium.*

**Figure 3:**
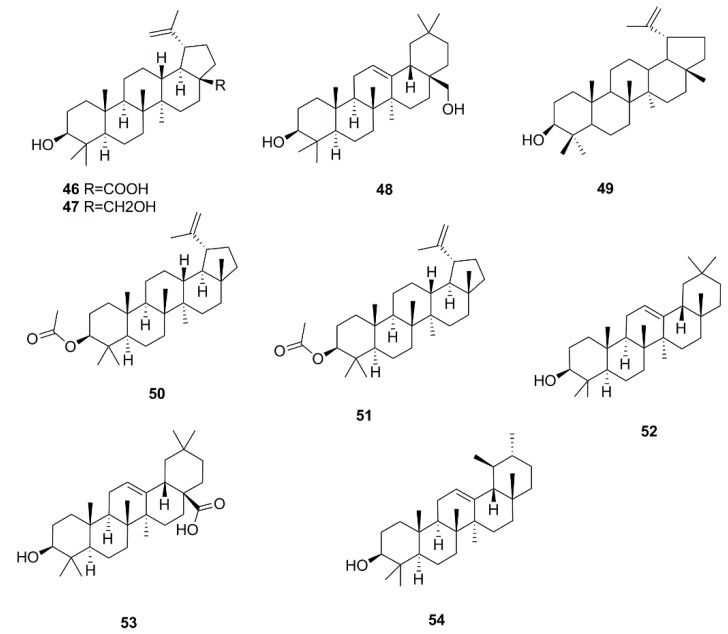
Chemical structures of the triterpenoids in *X. strumarium.*

**Figure 4:**
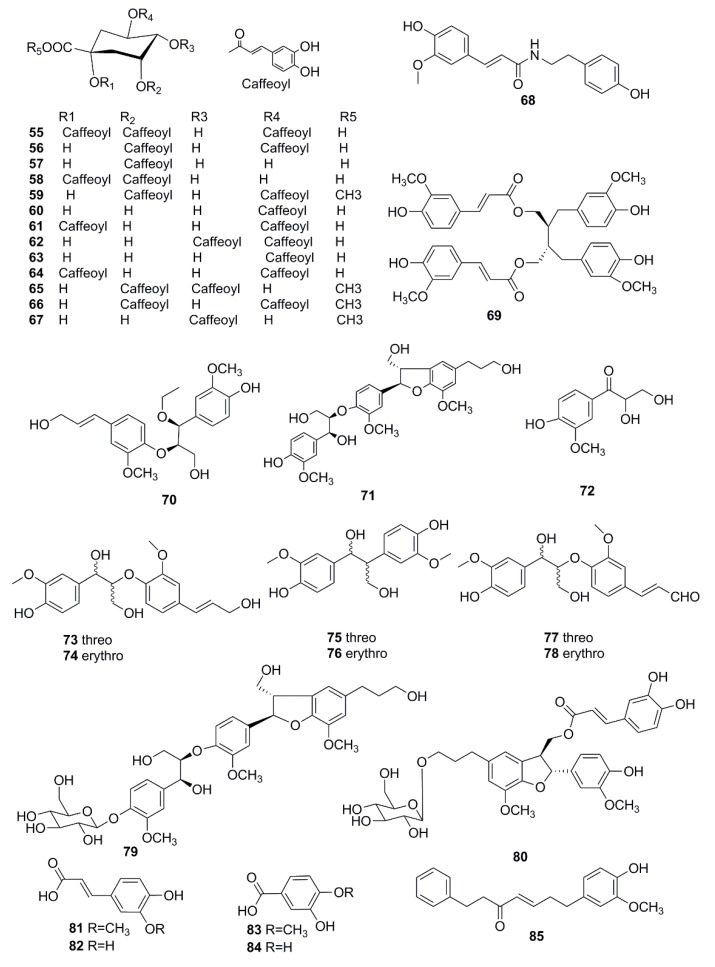

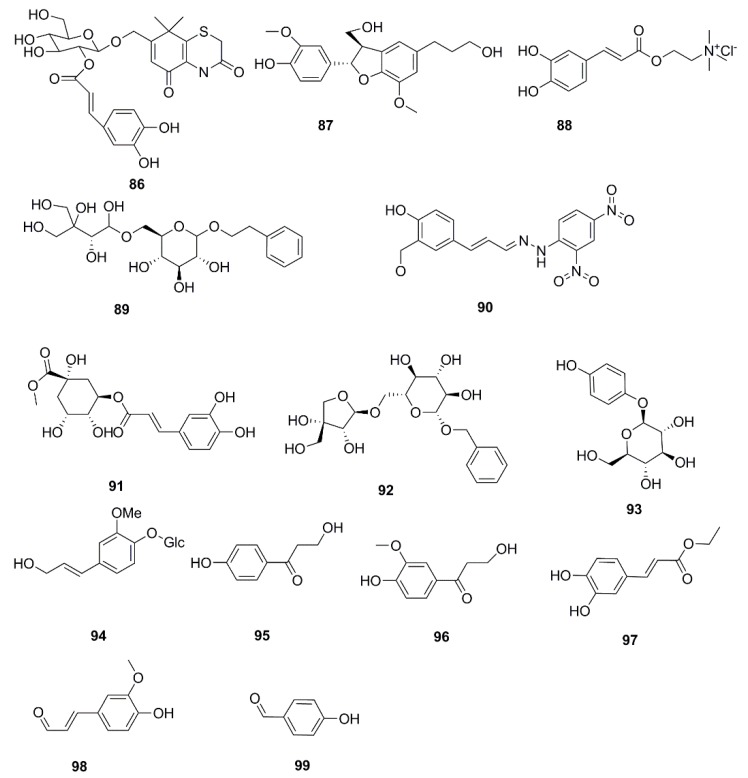
Chemical structures of the phenylpropenoids in *X. strumarium.*

**Figure 5:**
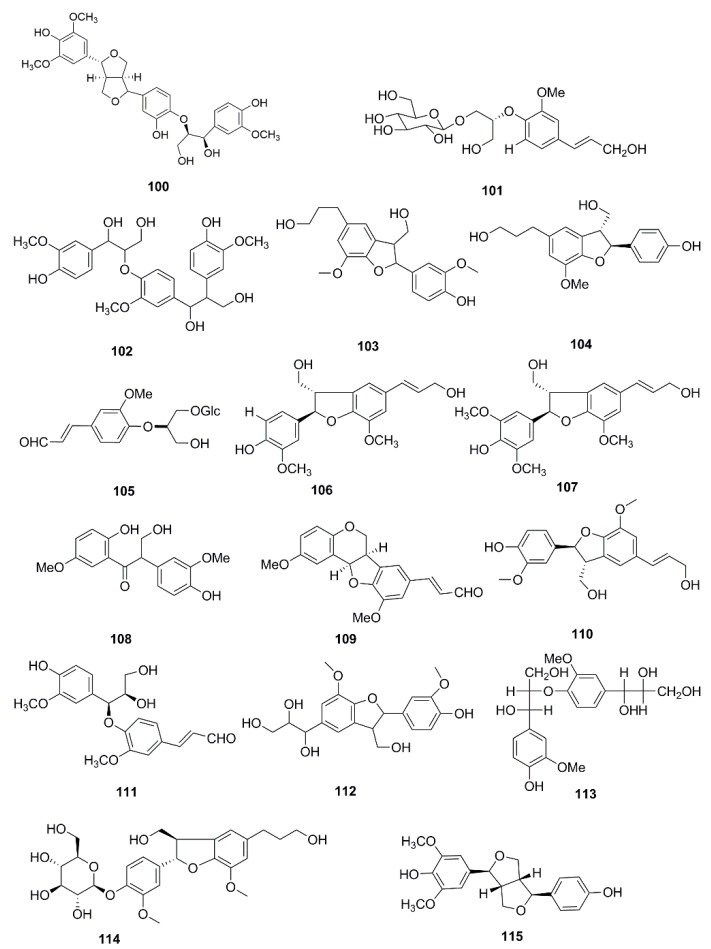
Chemical structures of the lignanoids in *X. strumarium.*

**Figure 6:**
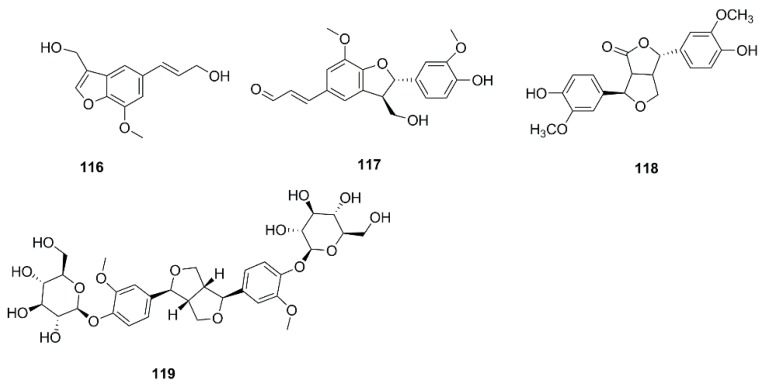
Chemical structures of the coumarins in *X. strumarium.*

**Figure 7:**
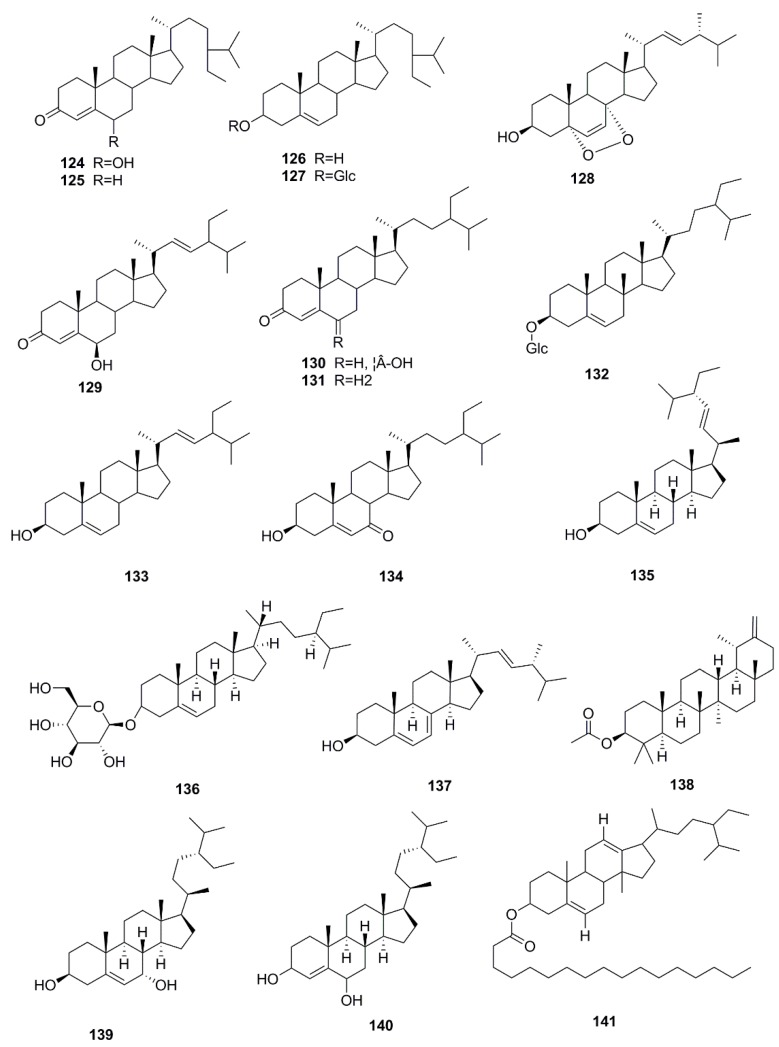
Chemical structures of the steroids in *X. strumarium.*

**Figure 8:**
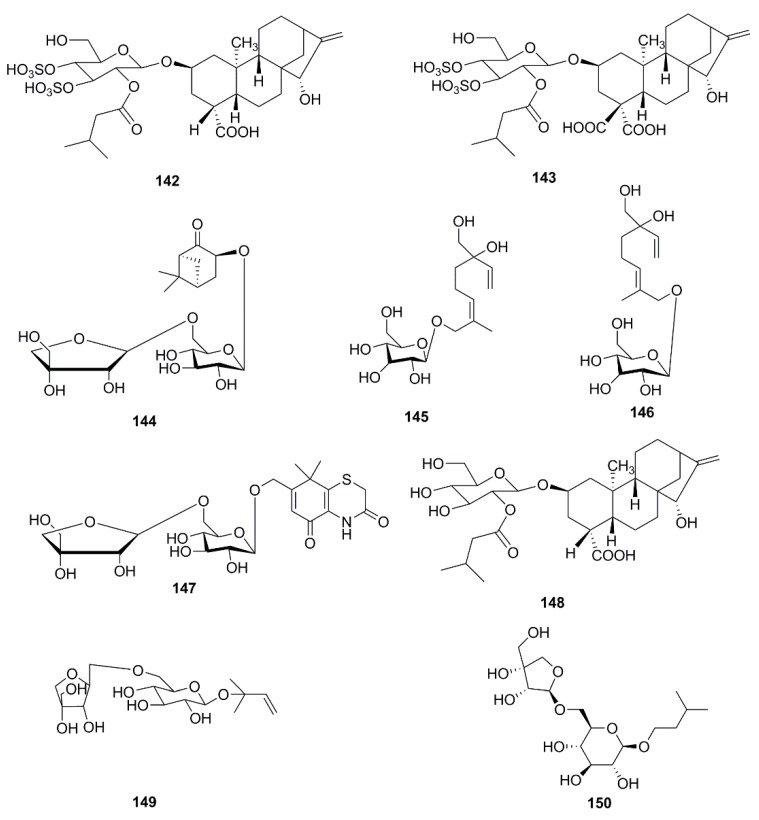
Chemical structures of the glycosides in *X. strumarium.*

**Figure 9:**
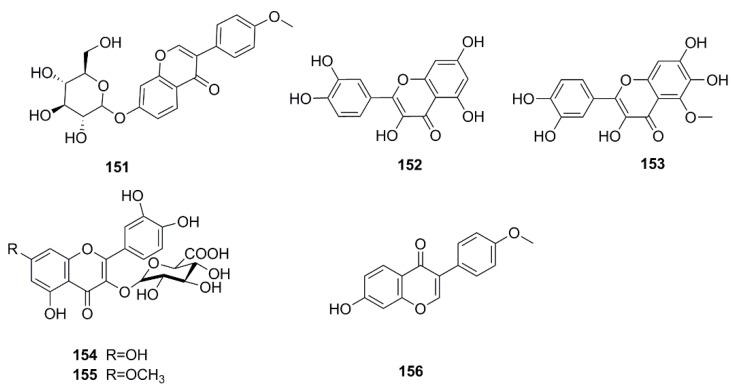
Chemical structures of the flavonoids in *X. strumarium.*

**Figure 10:**
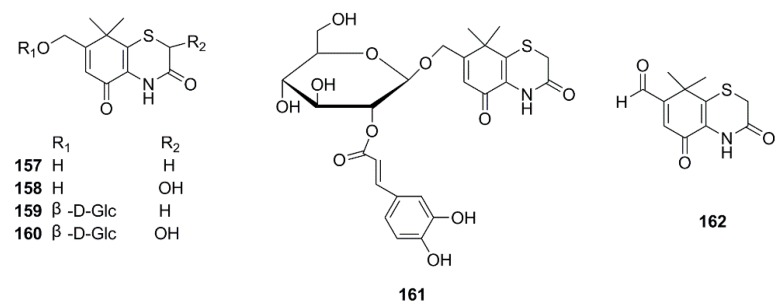
Chemical structures of the Thiazides in *X. strumarium.*

**Figure 11:**
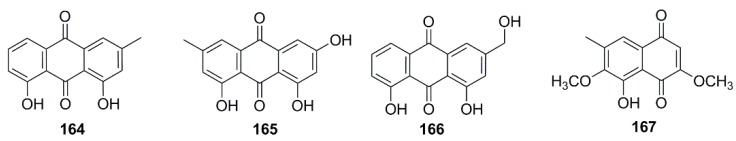
Chemical structures of the anthraquinones and naphthoquinones in *X. strumarium.*

**Figure 12:**
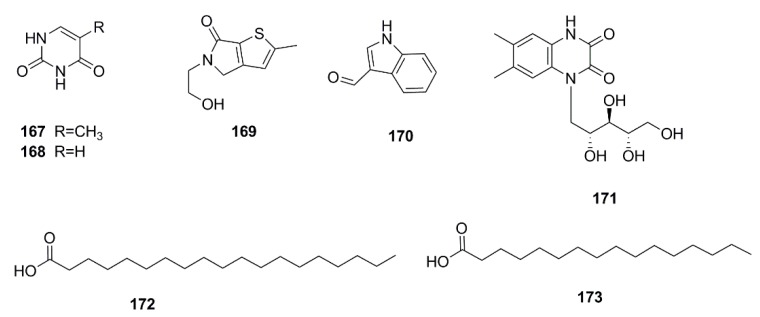
Chemical structures of other compounds in *X. strumarium.*

So far, many phytochemical studies of *X. strumarium* have been conducted, and more than 170 compounds have been isolated and identified from this plant. Among them, sesquiterpenes and phenylpropanoids are the most abundant and major bioactive constituents in *X. strumarium*, and are considered as the characteristic constituents of this plant. In addition to the chemical constituents found in fruits, constituents in other parts of *X. strumarium* were also comprehensively reported, including leaves, roots and stems, etc. In this section, the identified compounds are listed in the following table and the corresponding structures are also comprehensively presented. (Table 2, Figure 2, Figure 3, Figure 4, Figure 5, Figure 6, Figure 7, Figure 8, Figure 9, Figure 10, Figure 11 and Figure 12). 

### 4.1. Sesquiterpenoids and Triterpenoids

Sesquiterpenoids have many important biological functions and physiological activities, which are abundant in *X. strumarium*. Sesquiterpene lactones, the main characteristic components of plants in the Asteraceae family, exhibit strong activities with anti-microbial, antiviral, anti-tumor and anti-inflammation [55,56]. The predominant sesquiterpene lactones are the guaiane type and seco-guaiane type, of which xanthanolides are the important active constituent. In 2015, eight sesquiterpenes were isolated from the fruits of *X. strumarium*, including sibirolide A (**1**), sibirolide B (**2**) and norxanthantolide A–F (**3–8**) [13]. In addition, 1β-hydroxyl-5α-chloro-8-epi-xanthatin (**9**) and 11α, 13-dihydro-8-epi-xanthatin (**10**) were isolated from the aerial parts of *X. strumarium* [14]. Moreover, xanthinin (**11**), xanthumin (**12**), xanthanol (**13**), xanthanol acetate (**14**), isoxanthanol (**13**), xanthumanol (**16**), deacetoxylxanthumin (**17**), xanthatin (**18**), xanthinosin (**19**), tomentosin (**20**) were isolated from the leaves of *X. strumarium* [15,16]. Furthermore, other sesquiterpenoids were isolated and identified from the fruits, leaves and aerial parts of *X. strumarium*, including 8-epi-tomentosin (**21**) [17], 11α,13-dihydroxanthuminol (**22**), desacetylxanthanol (**23**) [18], (2E,4E,1’S,2’R,4’S,6’R)-dihydrophaseic acid (**24**) [19], 8-epi-xanthatin (**25**) [20], 2-hydroxy xanthinosin (**26**) [21], lasidiol *p*-methoxybenzoate (**27**) [18], 1β,4β, 4α,5α-diepoxyxanth-11(13)-en-12-oic acid (**28**), 11α,13-dihydroxanthatin (**29**), 4β, 5β-epoxyxanthatin-1α,4α-endoperoxide (**30**), 4-epi-xanthanol (**31**), 4-epi-isoxanthanol (**32**), 4-oxo-bedfordia acid (**33**) [22], 2-hydroxytomentosin (**34**), 2-hydroxytomentosin-1β,5β-epoxide (**35**) [20], xanthnon (**36**) [21], 6β,9β-dihydroxy-8-epi-xanthatin (**37**) [25], inusoniolide (**38**) [21], (3S,5R,6S,7E)-5,6-epoxy-3-hydroxy-7-megastigmene-9-one (**39**) [24], pungiolide E (**40**), pungiolide A (**41**), pungiolide D (**42**) [25], 5-azuleneacetic acid (**43**) [21], dihydrophaseic acid sodium salt 4’-O-β-d-glucopyranoside (**44**) [26], (3S,5R,6R,7E,9S)-megastigman-7ene-3,5,6,9-tetrol-3-O-β-d- glucopyranoside (**45**) [27]. 

Triterpenoids are another important kind of biomolecule found in *X. strumarium*. Nine triterpenoids including betulinic acid (**46**), botulin (**47**), erythrodiol (**48**) [28], lup-20(29)-en-3β-ol (**49**) [27], lupenyl acetate (**50**) [29], lupeol acetate (**51**) [30], β-amyrin (**52**), oleanolic acid (**53**) [31] and α-amyrin (**54**) [32] are reported from this plant. The chemical structures of these sesquiterpenoids and triterpenoids isolated from *X. strumarium* are shown in Figure 2 and Figure 3.

### 4.2. Phenylpropenoids

Phenylpropenoids are also important active constituents found in *X. strumarium*. To date, 45 phenylpropenoids have been reported in this plant. Phenolic acids, mainly chlorogenic acid, are considered to be the main anti-inflammatory and analgesic active ingredients and the highest content of organic acids [57]. The phenolic acids in *X. strumarium* contain caffeic acid, ferulic acid, and protocatechuic acid, etc. However, studies have shown that factors such as origin, harvesting time, processing time and temperature have obvious effects on the content of phenolic acid in *X. strumarium* [58]. Thirteen caffeoylquinic acids (CQA) derivatives were isolated from *X. strumarium*, including 1,3,5-tri-*O*-caffeoylquinic acid (**55**), 3,5-di-*O*-caffeoylquinic acid (**56**), neochlorogenic acid methyl ester (**57**), 1,3-di-*O*-caffeoylquinic acid (**58**), methyl-3,5-di-*O*-caffeoylquinic acid (**59**), chlorogenic acid (**60**), 1,4-di-*O*-caffeoylquinic acid (**61**), 4,5-di-*O*-caffeoylquinic acid (**62**), 5-*O*-caffeoylquinic acid (**63**), 1,5-di-*O*-caffeoylquinic acid (**64**), 3,4-di-caffeoylquinic acid methyl ester (**65**), 3,5-di-caffeoylquinic acid methyl ester (**66**), 4-*O*-caffeoyl quinic acid methyl ester (**67**) [33,34,35,36,37,38]. In addition, in 2017, N-trans-feruloyl tyramine (**68**) and 9,9’-*O*-di-(E)-feruloyl-(-)-secoisolariciresinol (**69**) were firstly reported in this plant [39].

Besides, some other phenylpropanoids were also isolated and identificated from this plan, such as xanthiumnolic A (**70**), xanthiumnolic C (**71**) [40], 2,3-dihydroxy-1-(4-hydroxy-3-methoxyphenyl)-propan-1-one (**72**) [41], threo-guaiacylglycerol-8-*O*-4’-(coniferyl alcohol) ether (**73**), erythro-guaiacylglycerol-8-*O*-4’-(coniferyl alcohol) ether (**74**), threo-1-phenyl-(4-hydroxy-3-methoxy)-2-phenyl-(4’’-hydroxy-3’’-methoxy)-1,3-propanediol (**75**), (1S,2R)-1,2-bis(4-hydroxy-3- methoxyphenyl)-1,3-propanediol (**76**), threo-guaiacylglycerol-β-coniferyl aldehyde ether (**77**), erythro-guaiacylglycerol-β-coniferyl aldehyde ether (**78**) [42], xanthiumnolic D (**79**), xanthiumnolic E (**80**) [40], ferulic acid (**81**) [43], caffeic acid (**82**) [36], protocatechuic acid (**83**) [19], isovanillic acid (**84**) [30], 7-(4-hydroxy-3-methoxyphenyl)-1-phenylhept-4-en-3-one (**85**) [28], xanthiazone-(2-*O*-caffeoyl)-β-d-glucopyranoside (**86**) [44], rel-(2α,3β)-7-*O*-methylcedrusin (**87**) [42], caffeic acid choline ester (**88**) [38], icariside D1 (**89**) [45], 3-methoxy-4-hydroxy-transcinnamaldehyde (**90**) [24], methylchlorogenate (**91**) [46], icariside F2 (**92**), arbutin (**93**), coniferine (**94**) [45], 3-hydoxy-1-(4-hydroxy-phenyl)-propan-1-one (**95**) [47], ω-hydroxypropioguaiacone (**96**) [45], caffeic acid ethyl ester (**97**) [19], 4-hydroxy-3-methoxycinnamaldehyde (**98**) [37], p-hydroxybenzaldehyde (**99**) [24], The chemical structures of these phenylpropenoids isolated from *X. strumarium* are shown in Figure 4.

### 4.3. Lignanoids and Coumarins

In recent years, some studies found that *X. strumarium* contain lignanoids and coumarins, moreover, 21 lignanoids and four coumarins have been discovered in this plant and are displayed in Figure 5 and Figure 6. In 2017, xanthiumnolic B (**100**) was found from the fruits of *X. strumarium* and its anti-inflammatory activity has been demonstrated [40]. Later, 14 lignanoids were also isolated from the fruits of *X. strumarium*, including (-)-1-*O*-β-d-glucopyranosyl-2-{2-methoxy-4-[1-(E)-propen-3-ol]phenoxyl} -propane-3-ol (**101**), leptolepisol D (**102**), dihydrodehydrodiconiferyl alcohol (**103**), chushizisin E (**104**), (-)-(2R)-1-*O*-β-d-glucopyranosyl-2-{2-methoxy-4-[(Eformylvinyl]phenoxyl}propane-3-ol (**105**), (-)-7*R*,8*S*-dehydrodiconiferyl alcohol (**106**), (-)-simulanol (**107**), 2-(4-hydroxy-3-methoxyphenyl)-3-(2-hydroxy-5-methoxyphenyl)-3-oxo-1-propanol (**108**), diospyrosin (**109**), dehydrodiconiferyl alcohol (**110**), balanophonin A (**111**), threo-dihydroxydehydrodiconiferyl alcohol (**112**), 1-(4-hydroxy-3-methoxy)-phenyl-2-[4-(1,2,3-trihydroxypropyl)-2-methoxy]-phenoxy-1,3-propandiol (**113**), 7*R*,8*S*-dihydrodehydrodiconiferyl alcohol 4-*O*-β-d-glucopyranoside (**114**) [48]. Furthermore, syringaresinol (**115**) [39], fructusol A (**116**) [42], balanophonin (**117**) [24], 4-oxopinoresinol (**118**) [28], pinoresinol (**119**) [24] were identified from the plant.

In 2011, Kan et al. isolated four coumarins from the roots of *X. strumarium* for the first time, including scopoletin (**120**), Jatrocin B (**121**), cleomiscosin A (**122**), cleomiscosin C (**123**) [39].

### 4.4. Steroids

A few studies have been conducted investigating the steroids in *X. strumarium*. In 2010, β-sitostenone (**124**), β-sitosterol (**125**), daucosterol (**126**), stigmast-4-en-β-ol-3-one (**127)**, and 5α,8α-epidioxy-22E-ergosta-6,22-dien-3β-ol (128) were isolated from *X. strumarium* [39]. Furthermore, Chen et al. found 6β-hydroxy-stigmast-4,22-dien-3-one (**129**), 6β-hydroxy-stigmast-4-en-3-one (**130**), 3-oxo-Δ4,5-sitostenone (**131**), β-daucosterol (**132**), β-stigmastero (**133**) and 7-ketositosterol (**134**) from the roots of *X. strumarium* [28]. 

Lately, stigmasterol (**135**), β-sitosterol-3-O-β-d-glucopyranoside (**136**) [31], ergosterol (**137**), taraxasteryl acetate (**138**) [30], 7α-hydroxy-β-sitosterol(stigmast-5-ene-3β,7α-diol) (**139**), stigmast-4-ene-3β,6α-diol (**140**) [24] and 14-methyl-12,13-dehydro-sitosterol-heptadeconate (**141**) [32] were isolated and identified in *X. strumarium*. The chemical structures of these steroids isolated from *X. strumarium* are shown in Figure 7.

### 4.5. Glycosides

In 1962, Song et al. isolated a toxic glycoside component named AA_2_ from the fruits of *X. strumarium*, which has been authenticated as atractyloside (**142**) by Wang in 1983 [49,59]. Subsequently, John et al. found another toxic ingredient known as carboxyatractyloside (**143**) in 1975 [50]. Research showed that the content of atractyloside in *X. strumarium* could be reduced after stir-flying, and its toxicity could be reduced. [60] Lately, seven other glycosides were separated from the fruits of *X. strumarium,* such as 3β-norpinan-2-one 3-*O*-β-d-apiofuranosyl-(1→6)-β-d-glucopyranoside (**144**), (6Z)-3-hydroxymethyl-7-methylocta-1,6-dien-3-ol 8-*O*-β-d-glucopyranoside (**145**), (6E)-3-hydroxymethyl-7-methylocta-1,6-dien-3-ol 8-*O*-β-d-glucopyranoside (**146**), 7-[(β-d-apiofuranosyl-(1→6)-β-d-glucopyranosyl)oxymethy]-8,8-dimethyl-4,8-dihydrobenzo[1,4]thiazine-3,5-dione (**147**) [41], 3’,4’-dedisulphated-atractyloside (**148**) [46], 2-methyl-3-buten-2-ol-phated-atractylosideimethy-d-glucopyranoside (**149**), everlastoside C (**150**) [51], and all glycosides are displayed in Figure 8.

### 4.6. Flavonoids

Flavonoids are common chemical components in plants all over the world. Six flavonoids including ononin (**151**) [43], quercetin (**152**), allopatuletin (**153**) [37], patuletin-3-glucuronide (**154**), quercetin-3-*O*-glucuronide (**155**) [34], formononetin (**156**) [43] have been isolated from this plant and are presented in Figure 9.

### 4.7. Thiazides

To this day, six thiazides from *X. strumarium* have been reported. In 1997, xanthiazone (**157**) was isolated from the aqueous acetone extract of the fruits [36]. Furthermore, 2-hydroxy-xanthiazone (**158**) [42], 7-hydroxymethyl-8,8-dimethyl-4,8-dihydrobenzol[1,4] thiazine-3,5-dione-11-*O*-β-d-glucopyranoside (**159**), 2-hydroxy-7-hydroxymethyl-8,8-dimethyl-4,8-dihydrobenzol[1,4]thiazine-3,5-dione-11-*O*-β-d-glucopyranoside (**160**) [43], 7-Hydroxymethyl-8,8-dimethyl-4,8-dihydrobenzol[1,4]thiazine-3,5-dione-(2-*O*-caffeoyl)-β-d-glucopyranoside (**161**) [52], and xanthialdehyde (**162**) [53] were identified from this plant (Figure 10).

A few studies have been focused on anthraquinones in *X. strumarium*. In one report in 2005, Huang et al. found chrysophanic acid (**163**), emodin (**164**) and aloe emodin (**165**) in the fruits of *X. strumarium* [54]. Then, the 5-hydroxy-3,6-dimethoxy-7-methyl-1,4-naphthalenedione (**166**), a new naphthoquinone, was isolated from the roots of *X. strumarium* [28] (Figure 11).

### 4.8. Other Compounds

Apart from these major types of phytochemical compounds mentioned above, there are some other chemical ingredients isolated from *X. strumarium*, including 5-methyluracil (**167**), uracil (**168**) [39], sibiricumthionol (**169**) [19], indole-3-carbaldehyde (**170**) [45], *N*-(1’-d-deoxyxylitolyl)-6,7-dimethyl-1,4-dihydro-2,3-quinoxalinedione (**171**) [38], nonadecanoic acid (**172**) [39], hexadecanoic acid (**173**) [32] (Figure 12). 

## 5. Pharmacology

### 5.1. Anti-AR Effect

*X. strumarium* is a traditional medicine widely used in the treatment of nasal diseases, especially allergic rhinitis (AR). In modern pharmacological study, the mechanism of *X. strumarium* in treating AR has been studied extensively. In 2003, it was reported that WEX inhibited compound 48/80 (C 48/80)-induced systemic anaphylaxis in mice (0.01 to 1 g/kg, p.o.), and the mechanism may be related to the inhibition of histamine and TNF-α released from rat peritoneal mast cells (RPMC) [61,62]. In 2008, Zhao et al. found that WEX (0.25–1 mg/mL) can modulate the human mast cell-mediated and peripheral blood mononuclear cell (PBMNC)-mediated inflammatory and immunological reactions which induced by pro-inflammatory cytokines including interleukin (IL)-4, IL-6, IL-8, GM-CSF and TNF-α [63]. Furthermore, the MEX is found to possess the inhibitory effect on the activation of C 48/80 stimulated mast cells, and the mechanism was correlated to inhibit Ca^2+^ uptake and histamine release, and increase cAMP in RPMC [64]. In addition, in 2014, Peng et al. demonstrated that the caffeoylxanthiazonoside (CXT) (5, 10, 20 mg/kg, p.o.) isolated from the fruits of *X. strumarium* was helpful to alleviate the nasal symptoms of ovalbumin (OVA) induced AR rats via anti-allergic, down-regulating IgE, anti-inflammatory and analgesic properties [65].

### 5.2. Anti-Tumor Effect

Anti-tumor effects are also regarded as primary pharmacological properties of *X. strumarium*, and have been extensively investigated in lung cancer, breast cancer, cervical cancer, colon cancer, liver cancer, meningioma, and leukemia.

Tao et al. studied the inhibitory effect of xanthatin (1–40 μM), an active agent in *X. strumarium*, against lung cancer cells (Cell lines of A549, H1975, H1299, H1650 and HCC827) and its potential mechanisms [66,67]. It found that xanthatin could downregulate the STAT3, GSK3β and β-catenin, moreover, xanthatin could also trigger Chk1-mediated DNA damage and destabilize Cdc25C via lysosomal degradation [66,67,68]. In 1995, Ahn et al. isolated three cytotoxic compounds from the leaves of *X. strumarium*, among them, xanthatin and 8-epi-xanthatin possessed obvious anti-tumor activity on A549 cells with IC_50_ (half maximal inhibitory concentration) values of 1.3 and 1.1 μg/mL, respectively [17]. Later, in 2002, it was reported that 1,8-epi-xanthatin epoxide has notable anti-tumor effect against A549 cells with IC_50_ value of 3.0 μM [69]. Furthermore, Wang et al. and Ferrer et al. reported that 8-epi-xanthatin-1α,5α-epoxide, 1β-hydroxyl-5α-chloro-8-epi-xanthatin and EEXA can inhibit the proliferation of A549 cells (IC_50_ = 9.5 μM, 20.7 μM and 52.2 μg/mL, respectively) [25,70]. 

In 2007, by using CellTiter 96 assay in vitro, Ramı’rez-Erosa et al. found that xanthatin and xanthinosin, two sesquiterpene lactones isolated from the burs of *X. strumarium*, obviously restrain the proliferation of breast cancer MDA-MB-231 cells with the IC_50_ values of 13.9 and 4.8 μg/mL, respectively [71]. Furthermore, Takeda et al. studied the mechanism of xanthatin against breast cancer MDA-MB-231 cells in 2011, and the results indicated that xanthatin (5–25 μM) inhibits cell growth via inducing caspase independent cell death which were irrelevant with FTase inhibition [72]. In addition, xanthatin (2.5–10 μM) can also up-regulate GADD45 γ tumor suppressor gene, and induce the prolonged expression of c-Fos via N-acetyl-l-cysteine-sensitive mechanism [73,74]. In 2016, the anti-tumor activity of EEXA on MFC7 cells was reported as well, with an IC_50_ value of 70.6 μg/mL [70]. 

In 2015, Vaishnav et al. demonstrated that WEX with a concentration of 12.5–50 μg/mL were able to induce death in HeLa cervical cancer cells by altering the antioxidant levels [75]. Recently, Liu et al. revealed that xanthatin (5–20 µM) targeted the selenocysteine (Sec) residue of thioredoxin reductase (TrxR) and inhibited the enzyme activity irreversibly [76]. Meanwhile, the inhibition of TrxR by xanthatin promoted oxidative stress-mediated apoptosis of HeLa cells.

In 1995, Ahn et al. reported that xanthatin and 8-epi-xanthatin were remarkably cytotoxic to colon cancer HCT-15 cells with ED_50_ (median effective dose) values of 1.1 and 0.1 μg/mL, respectively [17]. Later, in 2007, Ramı’rez-Erosa et al. (2007) found that xanthatin (IC_50_ = 6.15 μg/mL) and xanthinosin (IC_50_ = 6.15 μg/mL) possessed the function of inhibiting WiDr cells growth [71]. Furthermore, eremophil-1(10),11(13)-dien-12,8β-olide,8-epi-xanthatin-1β,5β-epoxide and tomentosin were isolated from the aerial parts of *X. strumarium*, and their anti-tumor activities on BGC-823 cells and KE-97 cells were aslo determined. The related results showed that the IC_50_ values of three compounds on BGC-823 cells are 13.22, 2.43, and 4.54 µM, respectively. Similarly, IC_50_ values of three compounds on BGC-823 cells are 4.41, 1.44, and 3.47 µM, respectively [77]. Moreover, Zhang et al. reported that xanthatin (3.9–18.6 µM) inhibited the proliferation of MKN-45 cells by inducing G2/M cell cycle arrest and apoptosis [78]. Later, in 2015, Karmakar et al. found that xanthinosin (8 µM) and lasidiol p-methoxybenzoate (16 µM) potentiate both extrinsic and intrinsic TRAIL-mediated apoptosis pathways and also decreased the level of cell survival protein Bcl-2 in AGS cells [20]. Simultaneously, fructusnoid C (IC_50_ = 7.6 µM) also reported to exhibit cytotoxic effects on AGS cells [79]. EEXA and CFEEXA have been identified as the active ingredients against the growth of CT26 cells with IC_50_ values of 58.9 and 25.3 μg/mL, respectively [70].

Furthermore, the anti-tumor effects of *X. strumarium* on liver cancers have also been reported in recent years. In 2013, Wang et al. found that the 1β-hydroxyl-5α-chloro-8-epi-xanthatin possessed significant in vitro cytotoxicity with an IC_50_ value of 5.1 µM against SNU387 cells [25]. Later, in 2017, the cytotoxic effects of MEX and EAFMEX on HepG2 cells were verified as LC_50_ (Lethal Concentration 50) values of 112.9 and 68.739 μg/mL [80]. Furthermore, Liu et al. demonstrated that xanthatin (5–40 μM) can induce HepG2 cells apoptosis by inhibiting thioredoxin reductase and eliciting oxidative stress [76].

Additionally, an investigation in 1995 indicated that Xanthatin and 8-epi-xanthatin both have cytotoxic effects on SK-MEL-2 cells with ED_50_ values 0.5 and 0.2 μg/mL, respectively [17]. In 2012, the EEXS showed notable inhibitory activity on Mel-Ab cells through downregulation of tyrosinase via GSK3β phosphorylation at concentrations of 1–50 μg/mL [81]. Later, in 2013, Li et al. reported the anti-tumor effects of xanthatin both in vitro and in vivo. Previous results showed that xanthatin (2.5–40 μM) possess a remarkable anti-proliferative effect against B16-F10 cells, and the related mechanism probably associated with activation of Wnt/β-catenin pathway as well as inhibition of angiogenesis. Meanwhile, the in vivo evidence in mice (xanthatin, 0.1–0.4 mg/10 g, i.p.) also verified the results mentioned above [82]. 

In 1994, DFEEXA was reported to be toxic to leukemia P-388 cells with an IC_50_ value of 1.64 μg/mL [83]. In addition, results of Nibret et al. showed that xanthatin has significant cytotoxic on HL-60 cells in 2011 [84]. Another report in 2017 reported that both MEX and EAFMEX have inhibitory effects on Jurkat cells, and EAFMEX showed higher toxicity to Jurkat cells when compared to MEX [80]. 

Besides, in 1995, Ahn et al. found that xanthatin and 8-epi-xanthatin have cytotoxic effects on CNS carcinoma XF-498 cells, and the ED_50_ values were 1.7 and 1.3 μg/mL, respectively [17]. In 2013, Pan et al. reported that WEX can cause significant cytotoxic effects on arcoma S180 cells in vivo (S180 cells bearing mice, 5–20 g/kg) [85]. The in vitro anti-proliferative activity of CEXR and MEXR on laryngeal cancer HEP-2 cells were implemented at doses of 12.5–100 µg/mL, and the two extracts of *X. strumarium* showed potent cytotoxic activities against the HEP-2 cells [86].

### 5.3. Anti-Inflammatory and Analgesic Effects

In 2004, it was reported that WEX (10, 100 and 1000 µg/mL) inhibited inflammatory responses in Lipopolysaccharide (LPS)-stimulated mouse peritoneal macrophages via decreasing IFN-γ, LPS-induced NO production and TNF-α production in a dose dependent manner [87]. Furthermore, in 2005, Kim et al. evaluated the anti-inflammatory and anti-nociceptive activities of MEX both in vitro and in vivo, it showed that the MEX (30, 60 and 90 mg/mL) can down-regulate the production of NO, PGE 2 and TNF-α, and MEX treatment (100 and 200 mg/kg/day, p.o.) clearly reduced carrageenan induced hind paw edema in rats [88]. In addition, MEX (100 and 200 mg/kg/day, p.o.) significantly reduced the amount of writhing induced by acetic acid, and increased jumping response latency in a hot plate test. Later, in 2008, xanthatin and xanthinosin were reported to inhibit LPS-induced inducible nitric oxide synthase and cyclooxygenase-2 (COX-2) expression in microglial BV-2 cells with IC_50_ values of 0.47 and 11.2 μM, respectively [89]. By using LPS inhibition assay and animal model of inflammation (carrageenan induced hind paw edema), the MEXL (100, 200 and 400 mg/kg) showed obvious anti-inflammatory activity both in vitro (IC_50_ = 87 μg/mL) and in vivo [90]. A report in 2015 showed that MEXR (50–400μg/mL) can suppress inflammatory responses via the inhibition of nuclear factor-κB (NF-κB) and signal transducer and activator of transcription 3 (STAT3) in LPS-induced murine macrophages [91]. Moreover, the WEX was found to restrain LPS-induced inflammatory responses through suppressing NF-κB activation, inhibiting JNK/p38 MAPK phosphorylation, and enhancing HO-1 expression in macrophages [92]. In 2016, Hossen et al. demonstrated that the inhibitory effect of MEX on the inflammatory disease possibly related to signaling inhibition of MAPK and AP-1 [93]. In another study, Hossen et al. found the potential anti-inflammatory activity of MEXA on LPS-treated macrophages and an HCl/EtOH-induced mouse model of gastritis by inhibiting PDK1 kinase activity and blocking signaling to its downstream transcription factor, NF-κB [94]. Later, in 2017, Jiang et al. found a new phenylpropanoid derivative named Xanthiumnolic E isolated from *X. strumarium*, which has notable inhibitory effect on LPS-induced nitric oxide (NO) production with IC_50_ value of 8.73 μM [26]. 

Additionally, *X. strumarium* was confirmed to inhibit some other kinds of inflammatory and painful diseases. In 2011, Huang et al. suggested that WEX inhibited the development of paw edema induced by carrageenan, and exhibited inhibitory activity on acetic acid effect and reduced the formalin effect at the late-phase (0.1, 0.5 and 1.0 g/kg, p.o.) [95]. In addition, the NFEEX at doses of 0.5, 0.75 and 1.0 mg/ear showed strong anti-inflammatory activity in the croton-oil-induced ear edema test, and reduced the amount of writhing induced by acetic acid in mice in a dose-dependent manner (100, 200 and 400 mg/kg) [96]. A report in 2011 demonstrated the anti-inflammatory activity of xanthatin by inhibiting both PGE 2 synthesis and 5-lipoxygenase activity at doses of 100 and 97 mg/mL, respectively [84]. Furthermore, Park et al. first explained the anti-inflammatory mechanism of EEX, which inhibited TNF-α/IFN-γ-induced expression of Th2 chemokines (TARC and MDC) by blocking the activation of the NF-κB, STAT1 and ERK-MAPK pathways in HaCaT keratinocytes [97]. The hot plate test, acetic acid induced writhing test and formalin test were applied to evaluate the analgesic activity of EEX, and it showed significant analgesic activity at concentrations of 250 and 500 mg/kg body weight [98].

### 5.4. Insecticide and Antiparasitic Effects

In 1995, Talakal et al. reported that EEXL possess anti-plasmodial activity against *Trypanosoma evansi* both in vitro and in vivo. The EEXL exhibited trypanocidal activity at all the four tested doses at 5, 50, 500 and 1000 µg/mL in vitro, and it can significantly prolong the survival period of the *T. evansi* infected mice at concentrations of 100, 300 and 1000 mg/kg [99]. In 2011, xanthatin was demonstrated to be the dominating insecticidal active compound against *Trypanosoma brucei brucei* with an IC_50_ value of 2.63mg/mL and a selectivity index of 20 [84]. In addition, Go¨kce et al. showed that MEX exhibited both ingestion toxicity and ovicidal activity to *Paralobesia viteana* with an LC_50_ of 11.02% (*w*/*w*) [100]. In 2012, by using schizont inhibition assay, the anti-plasmodial activity of EEXL against *Plasmodium berghei* was assessed, and it showed significant activity (IC_50_ = 4 µg/mL) and high selectivity index in vitro [101]. Later, in 2014, Roy et al. found that WEXL had distinct insecticidal properties against *Callosobruchus chinensis* with strong toxicity, repellent properties, inhibited fecundity and adult emergence of the insects at 1%, 2% and 4% concentrations [102]. Moreover, it is reported that EEX revealed anti-nematode activity against *Meloidogyne javanica* in inhibiting egg hatching and inducing mortality among second stage juveniles (J2s) [103]. Furthermore, the effect of MEX on the mortality rates of *Aedes caspius* and *Culex pipiens* were investigated, and the results revealed that the LC_50_ values of MEX were found to be 531.07 and 502.32 μg/mL against *A. caspius* and *C. pipiens*, respectively [80].

### 5.5. Antioxidant Effect

In 2010, it was reported that CEXR and MEXR showed significant free radical scavenging activity by 1,1-diphenyl-2-picrylhydrazyl (DPPH) method with LC_50_ values of 10.28 and 40.40 µg/mL, respectively [86]. After administration of PEEXW (250 and 500 mg/kg, p.o., for 20 days), the contents of superoxide dismutase, glutathione peroxidase, glutathione reductase and catalase significantly increased in rats’ brain [104]. Later, in 2011, Huang et al. found that WEX exhibited 70.6% to 76.4% and 35.2% to 79.1% scavenging activity on 2,2’-Azinobis-(3-ethylbenzthiazoline-6-sulphonate) (ABTS) radicals and DPPH radical scavenging in the concentration of 0.05–0.2 mg/mL; simultaneously, the reducing activity of WEX increased and liposome protection effect enhanced in a concentration-dependent manner with the same doses [95]. In the treatment with the MEXS (100 and 200 mg/kg, p.o. for 10 days), the contents of SOD, CAT, GSH and GPx were obviously increased in the diabetic rats’ tissues [105]. Moreover, in 2011, Sridharamurthy et al. evaluated the antioxidant effect of EEXR and CEXR by the scavenging activity of free radicals such as DPPH, super oxide, nitric oxide, and hydrogen peroxide [106]. Results showed that the IC_50_ values of EEXR were 29.81, 495.30, 395.20 and 10.18 µg/mL, respectively, and the IC_50_ values of CEXR were 24.85, 418.30, 415.18 and 9.23 µg/mL, respectively. In addition, Kamboj et al. demonstrated that EEXL possessed strong scavenging capacity against DPPH, nitric oxide and hydrogen peroxide with IC_50_ values of 85, 72 and 62 µg/mL. In addition, the antioxidant activity was possibly due to the presence of compounds in the extracts like flavonoid and phenolic [107]. In 2015, hexadecanoic acid, α-amyrin and 14-methyl-12,13-dehydro-sitosterol-heptadeconate were isolated from the leaves of *X. strumarium*, and their antioxidant potential was also evaluated. These three chemical components showed significant antioxidant activity in a dose dependent manner by DPPH and hydroxyl radical assay methods with the IC_50_ values of 106.4, 64.16, 76.18 µg/mL and 127.4, 83.96 and 84.4 µg/mL, respectively [32]. A study in 2017 revealed that the EOX displayed notable activity for DPPH radicals with an IC_50_ value of 138.87 μg/mL [108]. Furthermore, the antioxidant effects of the MEX obtained by the response surface methodology were measured by the scavenging activity towards the DPPH radical and Ferric ion reducing antioxidant power (FRAP). These results showed that methanol concentration and solid to solvent ratio were demonstrated to possess obvious effects on DPPH and FRAP values [28].

### 5.6. Antibacterial and Antifungal Effects

In 1983, Mehta et al. reported that the WEXFT possessed antimicrobial properties against *Vibrio cholera* [109]. Later, a study in 1997 revealed that the xanthatin isolated from the leaves of *X. strumarium* had notable potent activities against *Staphylococus epidermidis*, *Bacillus cereus*, *Klebsiella pneumoniae*, *Pseudomonas aeruginosa* and *Salmonella fyphi* with minimum inhibitory concentration (MIC) values of 31.3, 62.5, 31.3, 125 and 125 µg/mL, respectively [110]. In addition, it is reported that MEXL (500 and 100 mg/mL) exhibited strong activity against *K. pneumoniae*, *Proteus vulgaris*, *P. aeruginosa*, *Pseudomonas putida*, *Salmonella typhimurium*, *B. cereus, Bacillus subtilis and S. epidermidis* [111]. In 2015, Chen et al. also reported that β-sitosterol and β-daucosterol isolated from the *X. strumarium* have significant inhibitory effects against *Escherichia coli*, with MIC values of 0.17 and 0.35 mg/mL, respectively [112]. By using the disc diffusion method, Devkota et al. determined the antibacterial activity of MEXL and WEXL, and results showed that the two extracts inhibited growth towards *K. pneumoniae, Proteus mirabilis, E. coli, B. subtilis, Enterococcus faecalis* and *Staphylococcus aureus* at concentrations of 50, 100, 150, 200 and 250 mg/mL [113]. Moreover, Sharifi-Rad et al. revealed that EOXL can significantly suppress the growth of *S. aureus, B. subtilis, K. pneumoniae and P. aeruginosa* with MIC values of 0.5, 1.3, 4.8 and 20.5 µg/mL, respectively; additionally, EOXL (30, 60 and 120 mg/mL) also exhibited obvious antibacterial activity against Shiga toxin-producing *Escherichia coli* [114,115]. Furthermore, Wang et al. revealed that WEX possessed antibacterial potentials against *S. aureus* and *E. coli* with MIC values of 31.25 and 7.81 mg/mL, respectively [116]. Using the disk diffusion, the antibacterial activity of EOXF on *Rathayibacter toxicus* and *Pyricularia oryzae* was evaluated, and the MIC values were 25 and 12.5 µg/mL, respectively [108]. 

Similar to the antibacterial potentials, the antifungal activities of *X. strumarium* were also deeply investigated. In the year of 2002, Kim et al. found an antifungal constituent from *X. strumarium,* which was named deacetylxanthumin. It can inhibit mycelial growth and zoospore germination of *Phytophthora drechsleri* with a MIC value of 12.5 µg/mL [117]. In 2011, Yanar et al. used radial growth technique to test the antifungal activities of MEX against *Phytophthora infestans*, and the MEX showed the lowest MIC value of 2.0% *w/v* which was lower than the standard fungicide (Metalaxyl 4% + Mancuzeb 64%, MIC value was 2.5%, w/v) [118]. Later, in 2015, Sharifi-Rad et al. investigated the antifungal ability of EOXL on *Candida albicans* and *Aspergillus niger*, and the MIC values were 55.2 and 34.3 µg/mL, respectively [114]. In vitro, using the disk diffusion method, the EOXL exhibited strong inhibition against *Pyricularia oryzae* and *Fusarium oxysporum* with MIC values of 12.5 and 50 µg/mL, respectively [108]. Furthermore, the EOXL showed remarkable growth inhibition of a wide spectrum of fungal strains, such as *A. niger*, *Aspergillus flavus*, *F. oxysporum*, *Fusarium solani*, *Alternaria alternata* and *Penicillium digitatum* with both MIC and MBC (minimum bactericidal concentration) values of 8 µg/mL [119]. 

### 5.7. Antidiabetic Effect

In 1974, Kupiecki et al. found that the WEX (15 and 30 mg/kg, i.p.) exhibited potent hypoglycemic activity in normal rats in a dose-dependent manner [120]. In 2000, the antidiabetic effect of caffeic acid isolated from *X. strumarium* was investigated on both streptozotocin-induced and insulin-resistant rat models. The results showed that caffeic acid (0.5–3.0 mg/kg, i.v.) can decrease the plasma glucose level via increasing the glucose utilization [121]. In 2011, Narendiran et al. found that MEXS at the doses of 100 and 200 mg/kg (p.o., for 30 days) had remarkable diabetic activity in normal-glycemic and streptazocin induced hyperglycemic rats [105]. A report in 2013 demonstrated that the methyl-3,5-di-*O*-caffeoylquinate showed strong ability to counteract diabetic complications via competitive inhibition of aldose reductase (AR) and galactitol formation in rat lenses [47]. In addition, it is reported that the CFMEXL exhibited notable inhibitory activity on α-glucosidase enzyme with the IC_50_ value of 72 µg/mL [122]. Similarly, another study found that MEX also had a strong α-glucosidase inhibitory effect with IC_50_ value of 15.25 µg/mL [28].

### 5.8. Antilipidemic Effect

Recently, investigations into the antilipidemic effects of *X. strumarium* have been conducted. In 2011, the CEXR and EEXR were evaluated for anti-lipidemic activity in Triton WR-1339 induced hyperlipidemia in Swiss albino rats. The results showed that CEXR and EEXR (200 and 400 mg/kg p.o.) can significantly decrease the contents of plasma cholesterol, TG, LDL, and VLDL and increase plasma HDL levels, which was possiblely related to their significant antioxidant activity [106]. Later, in 2016, Li et al. found that WEX (570 and 1140 mg/kg, p.o., for 6 weeks) could improve the synthesis of fatty acid and TG, thus decreased the circulating free fatty acid (FFA) levels, indicating that WEX is involved in solving the abnormality of FFA in the circulation, which is executed by promoting the storage of the excess fat, rather than the elimination of added fat [123]. Furthermore, after treatment with WEX (3.7 and 11.11 g/kg, p.o., for 4 weeks), the blood glucose, TC, TG, LDLC levels decreased and HDLC levels increased in diabetic mice [124].

### 5.9. Antiviral Activity

In 2009, it was reported that the WEX (0.01, 0.1 and 1.0 g/kg, i.g., for 10 days) possessed antiviral activity against duck hepatitis B virus, and it can delay pathological changes [125]. In addition, five compounds were isolated from the fruits of *X. strumarium*, and their antiviral abilities were also evaluated. The results indicated that norxanthantolide F, 2-desoxy-6-epi-parthemollin, xanthatin, threo-guaiacylglycerol-8′-vanillic acid ether and caffeic acid ethyl ester exhibited notable activity against influenza A virus with IC_50_ values of 6.4, 8.6, 8.4, 8.4 and 3.7 µM, respectively by a cytopathic effect (CPE) inhibition method [13].

### 5.10. Other Pharmacological Effects

Apart from the pharmacological effects displayed above, *X. strumarium* also possesses some other activities. In 2016, the CXT (10, 20, and 40 mg/kg, i.p.) isolated from fruits of *X. strumarium* showed significant anti-septic activity in animal models of Cecal ligation and puncture (CLP) operation. Meanwhile, the CXT can increase survival rates of septic mice induced by CLP and decrease TNF-α and IL-6 levels induced by LPS in serum of mice [126]. After treatment with WEX (570 and 1140 mg/kg p.o., for 6 weeks), the glucose tolerance and insulin sensitivity improved, meanwhile, lipogenesis increases and lipid oxidation decreased in the liver of high-fat diet rats [127]. In 2014, Lin et al. demonstrated that the EEX (75 and 300 mg/kg, p.o.) can significantly inhibit paw swelling and arthritic score and increase body weight loss and decrease the thymus index in animal model of rheumatoid arthritis induced by Complete Freund’s Adjuvant (CFA) [128]. Moreover, the overproduction of TNF-α and IL-1β was notably suppressed in the serum of all EEX-treated rats. The anti-pyretic activity of MEXW (200 and 400 mg/kg, p.o.) was estimated on yeast induced hyperpyrexia, and it showed significant reduction in elevated body temperature [129]. Using Maximal Electroshock (MES) and Pentylenetetrazole (PTZ) induced seizures models, the anticonvulsant activity of PEEXW was tested, and results showed that PEEXW can reduce the mean duration of extensor phase and delay onset of myoclonic spasm and clonic convulsion of treated groups at doses of 250 and 500 mg/kg [130]. In 2016, Panigrah et al. explored the antiurolithiatic effect of HEEXB, and showed that HEEXB can restore the impairment induced by ethylene glycol including hyperoxaluria, crystalluria, hypocalciuria, polyurea, raised serum urea, creatinine, erythrocytic lipid peroxidise and nitric oxide, kidney calcium content as well as crystal deposition. The mechanism may be related to inhibition of various pathways involved in renal calcium oxalate formation, antioxidant property and down regulation of matrix glycoprotein, osteopontin (OPN) [131]. A report in 2012 indicated the antiulcer effect of EEXL in pylorus ligation induced gastric ulcers, and its gastro-protective mechanism may be due to DNA repair, free radical scavenging and down regulation of oxidativenitrosative stress along with cytokines [132]. In an in vivo study, with the CXT treatment (10, 20 and 40 mg/kg, p.o.), the cardiac hypertrophy reduced and fractional shortening (FS), ejection fraction (EF), cardiac output (CO) and heart rate (HR) reversed via suppressing the expression of pro-inflammatory cytokines and the NF-κB signaling pathway [133].

### 5.11. Summary of Pharmacologic Effects

In conclusion, *X. strumarium* has a wide range of pharmacological effects including anti-AR effects, anti-tumor effects, anti-inflammatory and analgesic effects, insecticide and antiparasitic effects, antioxidant effects, antibacterial and antifungal effects, antidiabetic effects, antilipidemic effects, and antiviral effects. (Table 3). It is noteworthy that the research areas of modern pharmacy primarily focus on chemical components and extracts, which indicated the promising potential of *X. strumarium* for treating disease. Nevertheless, the chemical constituents and corresponding pharmacological effects of *X. strumarium* are not systematically sorted out and analyzed. Therefore, it is necessary to investigate the pharmacological activity, structure-activity relationship and mechanism of *X. strumarium* both in vitro and in vivo experiments in the future.

## 6. Pharmacokinetics

Up to now, there are few reports on the pharmacokinetics of the extracts or monomers of *X. strumarium*. Previous pharmacokinetics studies of *X. strumarium* mainly focused on its active compounds including xanthatin, cryptochlorogenic acid, and toxic ingredient such as atractyloside. In 2014, a sensitive, specific and rapid ultra-high performance liquid chromatography (UHPLC) tandem mass spectrometry (UHPLC-MS/MS) method was applied to research pharmacokinetic properties of xanthatin in rat plasma. After intravenous injection of xanthatin at a dose of 2.4 mg/200 g, 4.8 mg/200 g and 9.6 mg/200 g, respectively. The t_1/2_ of three concentrations were found to be 108.58 ± 32.82, 123.50 ± 66.69, and 181.71 ± 148.26 min, respectively; and the peak plasma concentration (C_max_) values were 418.72 ± 137.51, 904.89 ± 193.53, and 1773.46 ± 1733.10 ng/mL, respectively. As the dose increased, the AUC_0–t_ and AUC_0–∞_ were gradually enlarged, and the AUC_0–t_ of three doses were 14,340.20 ± 7122.41, 32,149.52 ± 11,259.44, and 49,524.28 ± 28,520.88 n gh/mL, respectively; furthermore, the AUC_0–∞_ of three levels are 15,538.97 ± 7733.12, 36,431.22 ± 14,498.16, and 61,885.45 ± 30,704.80 n gh/mL, respectively. In addition, the total body CL were 0.13 ± 0.14, 0.17 ± 0.11, 0.22 ± 0.13 mL/min and V_d_ were 46.85 ± 20.19, 159.99 ± 30.49, and 208.22 ± 85.97 mL of three concentrations [134].

After intragastric administration of the atractyloside at doses of 11.4, 22.8, and 45.6 mg/kg, the peak time (T_max_) values were determined to be 0.38, 1.85, 0.27 h, respectively, the t_1/2_ were 13.64, 9.62, 8.61 h, respectively, and the peak plasma concentration (C_max_) values were 41.98, 24.61, 263.40 µg/mL, respectively. In addition, the area under the concentration-time curve (AUC) was also determined, and the AUC_0–t_ was 132.70, 222.90, and 345.20 µ gh/L. The results showed that the toxicokinetic behavior of atractyloside in rats was non-linear within the experimental dose range [135]. 

Furthermore, Shen et al. studied the pharmacokinetics of neochlorogenic acid and cryptochlorogenic acid in *X. strumarium* and its processed products after intragastric administration in rats. The results showed that the T_max_ of neochlorogenic acid and cryptochlorogenic acid in processed fruits of *X. strumarium* were 2.94 ± 0.18, and 3.00 ± 0.46 h, respectively; the t_1/2_ of neochlorogenic acid and cryptochlorogenic acid in processed fruits of *X. strumarium* were 2.35 ± 1.11, 1.97 ± 0.66 h. Moreover, the T_max_ of neochlorogenic acid and cryptochlorogenic acid in raw fruits of *X. strumarium* were 3.75 ± 0.46, 2.75 ± 0.27 h, and the t_1/2_ of neochlorogenic acid and cryptochlorogenic acid in raw fruits of *X. strumarium* were 1.70 ± 0.61, 2.12 ± 0.68 h. The neochlorogenic acid in fruits of *X. strumarium*, after being processed, takes effect quickly and lasts for a long time, while the cryptochlorogenic acid takes effect slowly and has a short action time [136].

## 7. Toxicity

In 1990, it was reported that *X. strumarium* has medium to strong allergenic effects and is poisonous to mammals, and atractyloside and carboxyatractyloside are considered to be the major toxic compounds [137]. *X. strumarium* is prudently ranked into the medium grade with less toxicity in the Shennong Bencao Jing, a monograph of materia medica. Some other Chinese materia medicas aslo record that *X. strumarium* possessed mild toxicity, such as Bencao Pinhui Jingyao, Bencao Huiyan. Thus, it is obvious that the ancient Chinese people have had a clear understanding of the toxicity of *X. strumarium* for a long time [138].

In recent years, many investigations have indicated the toxic effects and related mechanisms of the extracts and monomers of *X. strumarium* (Table 4). In 2005, Li et al. found that the median lethal concentration (LD_50_) value of the WEX in mice was 201.14 g/kg (i.g., crude herbs mass equal) [139]. In addition, a report in 2012 suggested that the LD_50_ value of the WEX in mice was 167.60 g/kg (crude herbs mass equal, i.g.), however the LD_50_ value was 194.15 g/kg (i.g., crude herb mass equivalent) in Fu’s research report [140,141]. These changes can be attributed to the toxicity of *X. strumarium* which varied with the processing method, genetic characteristics and growing conditions [138]. Furthermore, the LD_50_ value of the EEX in mice was 275.41 g/kg (crude herbs mass equal, i.g.), which was higher than WEX [140]. Another study showed that the carboxyatractyloside (10–100 mg, i.v.) can induce death in swine [142].

Recently, animal experiments and clinical studies on *X. strumarium* showed that hepatotoxicity is the main toxicity. In 2011, Wang et al. demonstrated that kaurene glycosides including atractylosid (50–200 mg/kg, i.p.) and carbxyatractyloside (50–150 mg/kg, i.p.) induced hepatotoxicity in mice by way of its induction of oxidative stress as lipid peroxidation in liver [143]. Besides, the chief mechanism of atractyloside poisoning is deemed to be inhibition of the mitochondrial ADP transporter [144]. Furthermore, the WFEEX and NFEEX (0.06, 0.3, 0.7 g/kg, i.g., for 28 days), which have marked hepatotoxicity to rats, can cause pathological changes, such as enlarged hepatic cell space, karyolysis, and inflammatory cell infiltration [145]. Moreover, it has been reported that WEX (21.0 g/kg i.g., for 28 days) significantly increased the content of ALT, AST in mice serum and decreased weight loss [146]. In addition, a study in 2014 found that WEX (7.5, 15.0 and 30.0 g/kg, i.g., for 5 days) can increased the serum ALT, AST, ALP, TBIL levels and the contents of LDL/vLDL, β-HB, glutamate, choline, acetate, glucose in male rats [147]. Finally, in 2018, Zeng et al. indicated that the contents of GLDH, α-GST increased and miRNA-122 decreased after administered WEX (16.7 g/kg i.g., for 7 days), which can be used as sensitive biomarkers for studying the regularity of hepatotoxicity of *X. strumarium* [148]. Apart from hepatotoxicity, Mandal et al. studied the neurotoxicity of the MEXA in mice and results show that MEXA (100, 200, 300 mg/kg) can obviously depress the action of central nervous system [149].

Many other studies have demonstrated that different medicinal parts and extraction parts are also cytotoxic to normal cells including hepatocytes, nephrocytes, ovary cells, etc. The cell inhibition ability of atractyloside on rat hepatocytes was investigated, and the results demonstrated that atractyloside (0.01–0.05 g/L) induced dose-dependent hepatotoxicity according to obvious decreases of cell viability, intracellular gluta-thione (GSH) content and albumin secretion [150]. Furthermore, atractyloside and carbxyatractyloside was reported to improve LDH activity and inhibit cell proliferation at the concentration of 100 μmol/L [147]. In 2013, Yu et al. indicated that WEX at concentrations 100 μg/mL can inhibit growth of HK-2 cells [151]. Moreover, HEXA (25–100 μg/mL) also causes in vitro DNA damage at cytotoxic concentrations through sister chromatid exchanges, chromosome aberrations, and comet assay, meanwhile, it also shows significant reduction in CHO cell viability [152]. In 2016, Su et al. compared the cytotoxicities of the components with different polarities, and study indicated that EAFEEX (IC_50_ = 231.1 μg/mL) was the most toxic part [153].

In recent years, few investigations have focused on the toxic effects of *X. strumarium* on reproduction. In 2014, it was reported that the WEX possessed reproductive toxicity to zebrafish embryos, including decreases in hatch rate, and increases in mortality rate, heart rate and swimming speed [154].

## 8. Future Perspectives and Conclusions

In summary, *X. strumarium*, which possesses anti-AR effects, anti-inflammatory and analgesic effects and anti-tumor effects, has been widely applied to clinical practice in many countries. In the meantime, many modern studies on *X. strumarium* were also carried out, and its pharmacological activities and chemical compositions have been preliminarily investigated. Nevertheless, how to find out the mechanism of pharmacological activities and its related compounds, develop clinical efficacy of *X. strumarium* and ensure medication safety are still extremely crucial now.

First, the chemical compounds and pharmacological activity studies of *X. strumarium* mainly focused on its fruits, but there are few investigations on the roots, leaves, stems and other parts of *X. strumarium*. In order to enlarge the source domain of the active compounds and maximize the plant utilization rate, it is very critical for researchers to conduct a comprehensive evaluation of other parts of this plant. Second, the fruits of *X. strumarium* are officially recognized as *Cang-Er-Zi* in the Chinese Pharmacopoeia (2015 Edition), but many other *Xanthium* species such as *X. mongolicum* Kitag, *Xanthium spinosum* L. and *Xanthium canadens* Mill were used as *X. strumarium* alternatives in many areas of China. Therefore, the physical properties, chemical compositions and pharmacological activities should be used to identify and differentiate the different varieties, and it is important to guarantee the safety and efficacy with these herbs to ensure its suitability for clinical use. Third, in China, *X. strumarium* is commonly used after processing in clinical medicine, but the mechanism of its detoxification still needs further study. The degree of processing depends mainly on the subjective experience of people, and it is difficult to ensure the consistency of the quality of Chinese Medicine. Thus, the intelligent sensory technology combined with artificial intelligence technology, such as machine vision, electronic nose and electronic tongue can be applied to standardize processing methods. Fourth, on the basis of current research progress in vivo and in vitro, many active compounds of *X. strumarium* have been found and identified, which are probably developed into effective drugs. Among them, xanthatin possessed strong anticancer activity against many kinds of tumors, which means that it has the potential to become an anticancer drug in the future. However, systematic investigations on pharmacokinetics, target-organ toxicity and clinical research of xanthatin will help to develop its bioactive constituents as novel drugs. Fifth, traditional Chinese medicine has the characteristics of multi-component, multi-target and multi-channel, and a single component cannot completely reveal its pharmacological activity. Recently, quality marker (Q-Markers) technologies have started to contribute to scientifically interpreting the correlation degree of effectiveness-material basis-quality control of significant components in traditional Chinese Medicine. For *X. strumarium*, Q-Markers technologies are able to clarify its possible action, toxicity mechanism and symbolic components, and it is helpful to establish the whole quality control and quality traceability system of *X. strumarium*.

## Figures and Tables

**Table 1:** The traditional and clinical uses of *Xanthium strumarium* in China.

Preparation Name	Main Compositions	Traditional and Clinical Uses	References
Li Bi Tablets	Xanthii Fructus, Scutellariae Radix, Magno1iae Flos, Menthae Haplocalycis Herba, Angelicae Dahuricae Radix, Asari Radix Et Rhizoma, Taraxaci Herba	Curing common cold with nasal obstruction, nasosinusitis, turbid nasal discharge	“Chinese Pharmacopoeia (2010)” ^a^
Shuang Xin Bi Dou Yan Ke Li	Xanthii Fructus, Magno1iae Flos, Angelicae Dahuricae Radix, Asari Radix Et Rhizoma, Lonicerae Japonicae Flos, Lonicerae Japonicae Cau1is, Taraxaci Herba, Glycyrrhizae Radix Et Rhizoma, Platycodonis Radix, Chrysanthemi Flos, Scutellariae Radix, Paeoniae Radix Rubra, Coicis Semen, Rehmanniae Radix	Treating nasosinusitis	“Guo Jia Zhong Cheng Yao Biao Zhun” ^b^
Xiao Er Bi Yan Tablets	Xanthii Fructus, Ligustici Rhizoma Et Radix, Saposhnikoviae Radix, Angelicae Dahuricae Radix, Polygoni Tinctorii Folium, Taraxaci Herba, Cimicifugae Rhizoma, Glycyrrhizae Radix Et Rhizoma	Curing chronic rhinitis of child	“Zhong Yao Cheng Fang Zhi Ji”^c^
Yu Yuan Wan	Xanthii Fructus, Scutellariae Radix, Gardeniae Fructus, Scrophulariae Radix, Magno1iae Flos, Ophiopogonis Radix, Lycii Cortex, Paeoniae Radix Rubra, Forsythiae Fructus, Angelicae Dahuricae Radix, Menthae Haplocalycis Herb, Schizonepetae Herba, Glycyrrhizae Radix Et Rhizoma, Platycodonis Radix	Treating redness and swelling of the nostrils, swelling and pain in throat	“Zhong Yao Cheng Fang Zhi Ji”^c^
Yi Xuan Ning Jiao Nang	Xanthii Fructus, Chrysanthemi Flos, Arisaema Cum Bile, Scutellariae Radix, Bambusae Caulis in Taenias, Ostreae Concha, Crataegi Fructus, Citri Reticulatae Pericarpium, Paeoniae Radix Alba Poria, Lycii Fructus	Treating hyperactivity of liver-yang, vertigo due to deficiency of Qi and blood	“Xin Yao Zhuan Zheng Biao Zhun” ^d^
Qing Re Zhi Ke Ke Li	Xanthii Fructus, Scutellariae Radix, Fritillariae Thunbergii Bulbus, Paridis Rhizoma, Commelinae Herba, Anemarrhenae Rhizoma, Gypsum Fibrosum, Citri Reticulatae Pericarpium, Aurantii Fructus, Armeniacae Semen Amarum, Platycodonis Radix	Curing cough, phlegm, fever, pharyngalgia, thirst, chest tightness, dry stool, yellow urine due to pulmonary retention of phlegmopyrexia; acute bronchitis, acute exacerbation of chronic bronchitis	“Xin Yao Zhuan Zheng Biao Zhun” ^d^
Di Tong Bi Yan Liquid	Xanthii Fructus, Taraxaci Herba, Asari Radix Et Rhizoma, Scutellariae Radix, Ephedrae Herba, Acori Tatarinowii Rhizoma, Angelicae Dahuricae Radix, Magno1iae Flos	Curing common cold with nasal obstruction, chronic rhinitis, allergic rhinitis, nasosinusitis	“Zhong Yao Cheng Fang Zhi Ji” ^c^
Di Tong Bi Yan Liquid Pen Wu Ji	Xanthii Fructus, Scutellariae Radix, Taraxaci Herba, Ephedrae Herba, Magno1iae Flos, Angelicae Dahuricae Radix, Asari Radix Et Rhizoma, Acori Tatarinowii Rhizoma	Curing common cold with nasal obstruction, chronic rhinitis, allergic rhinitis, nasosinusitis	“Xin Yao Zhuan Zheng Biao Zhun” ^d^
Fu Yang Chong Ji	Xanthii Fructus, Chuanxiong Rhizoma, Carthami Flos, Kochiae Fructus	Treating pruritus, eczema, urticaria	“Zhong Yao Cheng Fang Zhi Ji” ^c^
Dan Xiang Bi Yan Tablets	Xanthii Fructus, Pogostemonis Herba, Angelicae Dahuricae Radix, Centipedae Herba, Schizonepetae Herba, Lonicerae Japonicae Flos, Chrysanthemi Indici Flos	Curing chronic simple rhinitis, allergic rhinitis, acute and chronic rhinitis, and nasosinusitis	“Zhong Yao Cheng Fang Zhi Ji” ^c^
Nao Ning Tablets	Xanthii Fructus, Polygonati Rhizoma, Epimedii Folium, Ophiopogonis Radix, Ginseng Radix Et Rhizoma Rubra, Polygalae Radix, Ziziphi Spinosae Semen, Schisandrae Chinensis Fructus, Lycii Fructus, Cervi Cornu Pantotrichum, Testudinis Carapax Et Plastrum, Poria, Jujubae Fructus, Rehmanniae Radix Praeparata, Cervi Cornus Colla	Curing neurasthenia, forgetfulness and insomnia, dizziness and palpitation, weariness of body, weak health and spontaneous perspiration, impotence and spermatorrhea	“Zhong Yao Cheng Fang Zhi Ji” ^c^
Nao Ning Su Tablets	Xanthii Fructus, Polygonati Rhizoma, Lycii Fructus, Poria, Epimedii Folium, Polygalae Radix, Jujubae Fructus, Schisandrae Chinensis Fructus, Ziziphi Spinosae Semen, Ophiopogonis Radix, Testudinis Carapax Et Plastrum, Cervi Cornu Pantotrichum, Cervi Cornus Colla, Rehmanniae Radix Praeparata, Ginseng Radix Et Rhizoma	Curing neurasthenia, forgetfulness and insomnia, dizziness and palpitation, weariness of body, weak health and spontaneous perspiration, impotence and spermatorrhea	“Zhong Yao Cheng Fang Zhi Ji” ^c^
Qin Zhi Bi Yan Tang Jiang	Xanthii Fructus, Scutellariae Radix, Angelicae Dahuricae Radix, Ephedrae Herba, Magno1iae Flos, Centipedae Herba, Menthae Haplocalycis Herba	Treating acute rhinitis	“Chinese Pharmacopoeia (2015)” ^a^
Cang Yi Di Bi You	Xanthii Fructus, Angelicae Dahuricae Radix, Borneolum Syntheticum	Curing nasosinusitis, nasal obstruction and runny nose	“Zhong Yao Cheng Fang Zhi Ji”^c^
Cang Xin Qi Wu Ji	Xanthii Fructus, Magno1iae Flos, Asari Radix Et Rhizoma, Angelicae Dahuricae Radix, Coptidis Rhizoma	Curing nasal obstruction, rhinocnesmus, sneeze, allergic rhinitis, acute and chronic rhinitis	“Guo Jia Zhong Cheng Yao Biao Zhun” ^b^
Xin Yi Bi Yan Pills	Xanthii Fructus, Magno1iae Flos, Menthae Haplocalycis Herba, Perillae Folium, Glycyrrhizae Radix Et Rhizoma, Pogostemonis Herba, Centipedae Herba, Isatidis Radix, Angelicae Dahuricae Radix, Saposhnikoviae Radix, Houttuyniae Herba, Chrysanthemi Flos	Treating allergic rhinitis, chronic rhinitis, nervous headache, cold and rhinorrhea, nasal obstruction	“Zhong Yao Cheng Fang Zhi Ji” ^c^
Xin Qin Chong Ji	Xanthii Fructus, Asari Radix Et Rhizoma, Scutellariae Radix, Schizonepetae Herba, Saposhnikoviae Radix, Angelicae Dahuricae Radix, Astragali Radix, Atractylodis Macrocephalae Rhizoma, Cinnamomi Ramulus, Acori Tatarinowii Rhizoma	Curing allergic rhinitis due to deficiency of lung qi	“Zhong Yao Cheng Fang Zhi Ji” ^c^
Xin Qin Tablets	Xanthii Fructus, Asari Radix Et Rhizoma, Scutellariae Radix, Schizonepetae Herba, Saposhnikoviae Radix, Angelicae Dahuricae Radix, Astragali Radix, Atractylodis Macrocephalae Rhizoma, Cinnamomi Ramulus	Curing allergic rhinitis, deficiency of lung qi, exogenous pathogenic wind	“Xin Yao Zhuan Zheng Biao Zhun” ^d^
Xin Qin Ke Li	Xanthii Fructus, Asari Radix Et Rhizoma, Scutellariae Radix, Schizonepetae Herba, Saposhnikoviae Radix, Angelicae Dahuricae Radix, Astragali Radix, Atractylodis Macrocephalae Rhizoma, Cinnamomi Ramulus, Acori Tatarinowii Rhizoma	Curing rhinocnesmus, sneeze, rhinorrhea, cold, allergic rhinitis	“Chinese Pharmacopoeia (2010)” ^a^
Tong Qiao Bi Yan Tablets	Xanthii Fructus, Saposhnikoviae Radix, Astragali Radix, Magno1iae Flos, Atractylodis Macrocephalae Rhizoma, Menthae Haplocalycis Herba	Curing nasal obstruction, rhinorrhea, rhinocnesmus, forehead headache, chronic rhinitis, allergic rhinitis, nasosinusitis	“Chinese Pharmacopoeia (2010)” ^a^
Tong Qiao Bi Yan Jiao Nang	Xanthii Fructus, Saposhnikoviae Radix, Astragali Radix, Magno1iae Flos, Atractylodis Macrocephalae Rhizoma, Menthae Haplocalycis Herba	Curing nasal obstruction, rhinorrhea, rhinocnesmus, forehead headache, chronic rhinitis, allergic rhinitis, nasosinusitis	“Xin Yao Zhuan Zheng Biao Zhun” ^d^
Tong Qiao Bi Yan Ke Li	Xanthii Fructus, Astragali Radix, Magno1iae Flos, Menthae Haplocalycis Herba, Saposhnikoviae Radix, Angelicae Dahuricae Radix, Atractylodis Macrocephalae Rhizoma	Curing nasal obstruction, rhinocnesmus, rhinorrhea, forehead headache, chronic rhinitis, allergic rhinitis, nasosinusitis	“Chinese Pharmacopoeia (2015)” ^a^
Fang Zhi Bi Yan Tablets	Xanthii Fructus, Chrysanthemi Indici Flos, Centipedae Herba, Angelicae Dahuricae Radix, Saposhnikoviae Radix, Ecliptae Herba, Paeoniae Radix Alba, Arisaema Cum Bile, Glycyrrhizae Radix Et Rhizoma, Tribuli Fructus	Curing sneeze, nasal obstruction, headache, allergic rhinitis, nasosinusitis	“Zhong Yao Cheng Fang Zhi Ji”^c^
Bi Yan Qing Du Ji	Xanthii Fructus, Chrysanthemi Indici Flos, Paridis Rhizoma, Zanthoxyli Radix, Prunellae Spica, Gentianae Radix Et Rhizoma, Codonopsis Radix	Treating chronic inflammation of nasopharynx, swelling and pain in throat	“Zhong Yao Cheng Fang Zhi Ji”^c^
Bi Yan Qing Du Ke Li	Xanthii Fructus, Chrysanthemi Indici Flos, Paridis Rhizoma, Zanthoxyli Radix, Prunellae Spica, Gentianae Radix Et Rhizoma, Codonopsis Radix	Treating chronic inflammation of nasopharynx	“Chinese Pharmacopoeia (2015)” ^a^
Bi Yuan Pills	Xanthii Fructus, Magno1iae Flos, Lonicerae Japonicae Flos, Rubiae Radix Et Rhizoma, Chrysanthemi Indici Flos	Curing nasal obstruction, nasosinusitis, ventilation lack, rhinorrhea, anosmia, headache, pain of superciliary ridge	“Chinese Pharmacopoeia (2010)” ^a^
Bi Yuan He Ji	Xanthii Fructus, Magno1iae Flos, Lonicerae Japonicae Flos, Rubiae Radix Et Rhizoma, Chrysanthemi Indici Flos	Curing nasal obstruction, nasosinusitis, ventilation lack, rhinorrhea, anosmia, headache, pain of superciliary ridge	“Xin Yao Zhuan Zheng Biao Zhun” ^d^
Bi Yuan Tablets	Xanthii Fructus, Magno1iae Flos, Lonicerae Japonicae Flos, Rubiae Radix Et Rhizoma, Chrysanthemi Indici Flos	Curing chronic rhinitis, nasosinusitis	“Zhong Yao Cheng Fang Zhi Ji”^c^
Bi Yuan Shu Kou Fu Ye	Xanthii Fructus, Magno1iae Flos, Menthae Haplocalycis Herba, Angelicae Dahuricae Radix, Scutellariae Radix, Gardeniae Fructus, Bupleuri Radix, Asari Radix Et Rhizoma, Chuanxiong Rhizoma, Astragali Radix, Clematidis Armandii Caulis, Platycodonis Radix, Poria	Curing rhinitis, nasosinusitis	“Chinese Pharmacopoeia (2010)” ^a^
Bi Yuan Shu Jiao Nang	Xanthii Fructus, Magno1iae Flos, Menthae Haplocalycis Herba, Angelicae Dahuricae Radix, Scutellariae Radix, Gardeniae Fructus, Bupleuri Radix, Asari Radix Et Rhizoma, Chuanxiong Rhizoma, Astragali Radix, Clematidis Armandii Caulis, Platycodonis Radix, Poria	Curing rhinitis, nasosinusitis	“Chinese Pharmacopoeia (2010)” ^a^
Bi Yuan Tong Qiao Ke Li	Xanthii Fructus, Magno1iae Flos, Ephedrae Herba, Angelicae Dahuricae Radix, Menthae Haplocalycis Herba, Ligustici Rhizoma Et Radix, Scutellariae Radix, Forsythiae Fructus, Chrysanthemi Indici Flos, Trichosanthis Radix, Rehmanniae Radix, Salviae Miltiorrhizae Radix Et Rhizoma, Poria, Glycyrrhizae Radix Et Rhizoma	Curing acute nasosinusitis, nasal obstruction, headache, fever	“Chinese Pharmacopoeia (2015)” ^a^
Bi Yan Ling Pills	Xanthii Fructus, Magno1iae Flos, Angelicae Dahuricae Radix, Asari Radix Et Rhizoma, Scutellariae Radix, Menthae Haplocalycis Herba, Fritillariae Cirrhosae Bulbus, Sojae Semen Praeparatum	Curing nasosinusitis, nasal obstruction, chronic rhinitis	“Zhong Yao Cheng Fang Zhi Ji” ^c^
Bi Yan Ling Tablets	Xanthii Fructus, Magno1iae Flos, Angelicae Dahuricae Radix, Asari Radix Et Rhizoma, Scutellariae Radix, Fritillariae Cirrhosae Bulbus, Sojae Semen Praeparatum	Treating chronic nasosinusitis, rhinitis, nasal obstruction and headache, anosmia	“Zhong Yao Cheng Fang Zhi Ji” ^c^
Bi Yan Tablets	Xanthii Fructus, Magno1iae Flos, Saposhnikoviae Radix, Forsythiae Fructus, Chrysanthemi Indici Flos, Schisandrae Chinensis Fructus, Platycodonis Radix, Angelicae Dahuricae Radix, Anemarrhenae Rhizoma, Schizonepetae Herba, Glycyrrhizae Radix Et Rhizoma, Phellodendri Chinensis Cortex, Ephedrae Herba, Asari Radix Et Rhizoma	Treating acute and chronic rhinitis, nasal obstruction, rhinorrhea, fever, headache	“Chinese Pharmacopoeia (2010)” ^a^
Bi Yan Tang Jiang	Xanthii Fructus, Scutellariae Radix, Angelicae Dahuricae Radix, Ephedrae Herba, Magno1iae Flos, Centipedae Herba, Menthae Haplocalycis Herba	Treating acute rhinitis	“Zhong Yao Cheng Fang Zhi Ji” ^c^
Bi Dou Yan Kou Fu Yan	Xanthii Fructus, Magno1iae Flos, Menthae Haplocalycis Herba, Platycodonis Radix, Bupleuri Radix, Angelicae Dahuricae Radix, Chuanxiong Rhizoma, Scutellariae Radix, Gardeniae Fructus, Poria, Clematidis Armandii Caulis, Astragali Radix	Curing nasal obstruction due to wind-heat affecting lung, acute and chronic rhinitis, nasosinusitis	“Chinese Pharmacopoeia (2010)” ^a^
Bi Shu Shi Tablets	Xanthii Fructus, Chrysanthemi Indici Flos, Centipedae Herba, Angelicae Dahuricae Radix, Saposhnikoviae Radix, Ecliptae Herba, Paeoniae Radix Alba, Arisaema Cum Bile, Glycyrrhizae Radix Et Rhizoma, Tribuli Fructus	Curing sneeze, rhinorrhea, nasal obstruction, headache, allergic rhinitis, chronic nasosinusitis	“Zhong Yao Cheng Fang Zhi Ji” ^c^
Bi Tong Pills	Xanthii Fructus, Magno1iae Flos, Angelicae Dahuricae Radix, Centipedae Herba, Menthae Haplocalycis Herba, Scutellariae Radix, Glycyrrhizae Radix Et Rhizoma	Curing affection of exogenous wind-heat, chronic rhinitis	“Zhong Yao Cheng Fang Zhi Ji” ^c^

^a^ Cited from “Chinese Pharmacopoeia”; ^b^ Cited from “Guo Jia Zhong Cheng Yao Biao Zhun”; ^c^ Cited from “Zhong Yao Cheng Fang Zhi Ji”; ^d^ Cited from “Xin Yao Zhuan ZhengBiao Zhun”. Xanthii Fructus means the fruits of *Xanthium strumarium* L.

**Table 2:** Chemical constituents isolated from *X. strumarium*.

Classification	No.	Chemical Component	Part of Plant	Reference
Sesquiterpenoids	1	sibirolide A	Fruits	[13]
	2	sibirolide B	Fruits	[13]
	3	norxanthantolide A	Fruits	[13]
	4	norxanthantolide B	Fruits	[13]
	5	norxanthantolide C	Fruits	[13]
	6	norxanthantolide D	Fruits	[13]
	7	norxanthantolide E	Fruits	[13]
	8	norxanthantolide F	Fruits	[13]
	9	1β-hydroxyl-5α-chloro-8-epi-xanthatin	Aerial parts	[14]
	10	11α,13-dihydro-8-epi-xanthatin	Aerial parts	[14]
Sesquiterpenoids	11	xanthinin	Leaves	[15]
	12	xanthumin	Leaves	[15]
	13	xanthanol	Leaves	[15]
	14	xanthanol Acetate	Leaves	[15]
	15	isoxanthanol	Leaves	[15]
	16	xanthumanol	Leaves	[16]
	17	deacetoxylxanthumin	Leaves	[16]
	18	xanthatin	Leaves	[16]
	19	xanthinosin	Leaves	[16]
	20	tomentosin	Leaves	[16]
	21	8-epi-tomentosin	Leaves	[17]
	22	11α,13-dihydroxanthuminol	Leaves	[18]
	23	desacetylxanthanol	Leaves	[18]
	24	(2E,4E,1’S,2’R,4’S,6’R)-dihydrophaseic acid	Fruits	[19]
	25	8-epi-xanthatin	Aerial parts	[20]
	26	2-hydroxy xanthinosin	Aerial parts	[21]
	27	lasidiol p-methoxybenzoate	Leaves	[18]
	28	1β, 4β, 4α,5α-diepoxyxanth-11(13)-en-12-oic acid	Aerial parts	[22]
	29	11α,13-dihydroxanthatin	Aerial parts	[22]
	30	4β,5β-epoxyxanthatin-1α,4α-endoperoxide	Aerial parts	[22]
	31	4-epi-xanthanol	Aerial parts	[22]
	32	4-epi-isoxanthanol	Aerial parts	[22]
	33	4-oxo-bedfordia acid	Aerial parts	[22]
	34	2-hydroxytomentosin	Aerial parts	[20]
	35	2-hydroxytomentosin-1β,5β-epoxide	Aerial parts	[20]
	36	xanthnon	Aerial parts	[21]
	37	6β,9β-dihydroxy-8-epi-xanthatin	Leaves	[23]
	38	inusoniolide	Aerial parts	[21]
	39	(3S,5R,6S,7E)-5,6-epoxy-3-hydroxy-7-megastigmene-9-one	Fruits	[24]
	40	pungiolide E	Aerial parts	[25]
	41	pungiolide A	Aerial parts	[25]
	42	pungiolide D	Aerial parts	[25]
	43	5-azuleneacetic acid	Aerial parts	[21]
	44	dihydrophaseic acid sodium salt 4’-*O*-β-d-glucopyranoside	Fruits	[26]
	45	(3S,5R,6R,7E,9S)-megastigman-7ene-3,5,6,9-tetrol-3-*O*-β-d-glucopyranoside	Aerial parts	[27]
Triterpenoids	46	betulinic acid	Roots	[28]
	47	betulin	Roots	[28]
	48	erythrodiol	Roots	[28]
	49	lup-20(29)-en-3β-ol	Aerial parts	[27]
Triterpenoids	50	lupenyl acetate	Aerial parts	[29]
	51	lupeol acetate	Whole plants	[30]
	52	β-amyrin	Aerial parts	[31]
	53	oleanolic acid	Aerial parts	[31]
	54	α-amyrin	Leaves	[32]
Phenylpropenoids	55	1,3,5-tri-*O*-caffeoylquinic acid	Fruits	[33]
	56	3,5-di-*O*-caffeoylquinic acid	Fruits	[33]
	57	neochlorogenic acid methyl ester	Fruits	[34]
	58	1,3-di-*O*-caffeoylquinic acid	Fruits	[34]
	59	methyl-3,5-di-*O*-caffeoylquinic acid	Fruits	[34]
	60	chlorogenic acid	Fruits	[35]
	61	1,4-di-*O*-caffeoylquinic acid	Fruits	[35]
	62	4,5-di-*O*-caffeoylquinic acid	Fruits	[35]
	63	5-*O*-caffeoylquinic acid	Fruits	[35]
	64	1,5-di-*O*-caffeoylquinic acid	Fruits	[36]
	65	3,4-di-caffeoylquinic acid methyl ester	Fruits	[37]
	66	3,5-di-caffeoylquinic acid methyl ester	Fruits	[37]
	67	4-*O*-caffeoyl quinic acid methyl ester	Fruits	[38]
	68	N-trans-feruloyl tyramine	Roots	[39]
	69	9,9’-*O*-di-(E)-feruloyl-(-)-secoisolariciresinol	Roots	[39]
	70	xanthiumnolic A	Fruits	[40]
	71	xanthiumnolic C	Fruits	[40]
	72	2,3-dihydroxy-1-(4-hydroxy-3-methoxyphenyl)-propan-1-one	Fruits	[41]
	73	threo-guaiacylglycerol-8-*O*-4’- (coniferyl alcohol) ether	Fruits	[42]
	74	erythro-guaiacylglycerol-8-*O*-4’-(coniferyl alcohol) ether	Fruits	[42]
	75	threo-1-phenyl-(4-hydroxy-3-methoxy)-2-phenyl-(4’’-hydroxy-3’’-methoxy)-1,3-propanediol	Fruits	[42]
	76	(1S,2R)-1,2-bis(4-hydroxy-3-methoxyphenyl)-1,3-propanediol	Fruits	[42]
	77	threo-guaiacylglycerol-β-coniferyl aldehyde ether	Fruits	[42]
	78	erythro-guaiacylglycerol-β-coniferyl aldehyde ether	Fruits	[42]
	79	xanthiumnolic D	Fruits	[40]
	80	xanthiumnolic E	Fruits	[40]
	81	ferulic acid	Fruits	[43]
	82	caffeic acid	Fruits	[36]
	83	protocatechuic acid	Fruits	[19]
	84	isovanillic acid	Whole plants	[30]
	85	7-(4-hydroxy-3-methoxyphenyl)-1-phenylhept-4-en-3-one	Roots	[28]
Phenylpropenoids	86	xanthiazone-(2-*O*-caffeoyl)-β-d-glucopyranoside	Whole plants	[44]
	87	rel-(2α,3β)-7-*O*-methylcedrusin	Fruits	[42]
	88	caffeic acid choline ester	Fruits	[38]
	89	icariside D1	Fruits	[45]
	90	3-methoxy-4-hydroxy-transcinnamaldehyde	Fruits	[24]
	91	methylchlorogenate	Fruits	[46]
	92	icariside F2	Fruits	[45]
	93	arbutin	Fruits	[45]
	94	coniferine	Fruits	[45]
	95	3-hydoxy-1-(4-hydroxy-phenyl)-propan-1-one	Fruits	[47]
	96	ω-hydroxypropioguaiacone	Fruits	[45]
	97	caffeic acid ethyl ester	Fruits	[19]
	98	4-hydroxy-3-methoxycinnamaldehyde	Fruits	[37]
	99	p-hydroxybenzaldehyde	Fruits	[24]
Lignanoids	100	xanthiumnolic B	Fruits	[40]
	101	(-)-1-*O*-β-d-glucopyranosyl-2-{2-methoxy-4-[1-(E)-propen-3-ol]phenoxyl}-propane-3-ol	Fruits	[48]
	102	leptolepisol D	Fruits	[48]
	103	dihydrodehydrodiconiferyl alcohol	Fruits	[48]
	104	chushizisin E	Fruits	[48]
	105	(-)-(2R)-1-*O*-β-d-glucopyranosyl-2-{2-methoxy-4-[(E)formylviny1]phenoxyl}propane-3-ol	Fruits	[48]
	106	(-)-7R,8S-dehydrodiconiferyl alcohol	Fruits	[48]
	107	(-)-simulanol	Fruits	[48]
	108	2-(4-hydroxy-3-methoxyphenyl)-3-(2-hydroxy-5-methoxyphenyl)-3-oxo-1-propanol	Fruits	[48]
	109	diospyrosin	Fruits	[48]
	110	dehydrodiconiferyl alcohol	Fruits	[48]
	111	balanophonin A	Fruits	[48]
	112	threo-dihydroxydehydrodiconiferyl alcohol	Fruits	[48]
Lignanoids	113	1-(4-hydroxy-3-methoxy)-phenyl-2-[4-(1,2,3-trihydroxypropyl)-2-methoxy]-phenoxy-1,3-propandiol	Fruits	[48]
	114	7R,8S-dihydrodehydrodiconiferyl alcohol 4-*O*-β-d-glucopyranoside	Fruits	[48]
	115	syringaresinol	Roots	[39]
	116	fructusol A	Fruits	[42]
	117	balanophonin	Fruits	[24]
	118	4-oxopinoresinol	Roots	[28]
	119	pinoresinol	Fruits	[24]
Coumarins	120	jatrocin B	Roots	[39]
Coumarins	121	cleomiscosin A	Roots	[39]
	122	cleomiscosin C	Roots	[39]
	123	scopoletin	Roots	[39]
Steroids	124	stigmast-4-en-β-ol-3-one	Roots	[39]
	125	β-sitostenone	Roots	[39]
	126	β-sitosterol	Fruits, Leaves	[39]
	127	daucosterol	Fruits	[39]
	128	5α,8α-epidioxy-22E-ergosta-6,22-dien-3β-ol	Roots	[39]
	129	6β-hydroxy-stigmast-4,22-dien-3-one	Roots	[28]
	130	6β-hydroxy-stigmast-4-en-3-one	Roots	[28]
	131	3-oxo-△^(4,5)^-sitostenone	Roots	[28]
	132	β-daucosterol	Roots	[28]
	133	β-stigmasterol	Roots	[28]
	134	7-ketositosterol	Roots	[28]
	135	stigmasterol	Aerial parts	[31]
	136	β-sitosterol-3-*O*-β-d-glucopyranoside	Aerial parts	[31]
	137	ergosterol	Whole plants	[30]
	138	taraxasteryl acetate	Whole plants	[30]
	139	7α-hydroxy-β-sitosterol (stigmast-5-ene-3β,7α-diol)	Fruits	[24]
	140	stigmast-4-ene-3β,6α-diol	Fruits	[24]
	141	14-methyl-12,13-dehydro-sitosterol-heptadeconate	Leaves	[32]
Glycosides	142	atractyloside	Fruits	[49]
	143	carboxyatractyloside	Burrs	[50]
	144	3β-norpinan-2-one 3-*O*-β-d-apiofuranosyl-(1→6)-β-d-glucopyranoside	Fruits	[41]
	145	(6Z)-3-hydroxymethyl-7-methylocta-1,6-dien-3-ol 8-*O*-β-d-glucopyranoside	Fruits	[41]
	146	(6E)-3-hydroxymethyl-7-methylocta-1,6-dien-3-ol 8-*O*-β-d-glucopyranoside	Fruits	[41]
	147	7-[(β-d-apiofuranosyl-(1→6)-β-d-glucopyranosyl)oxymethy]-8,8-dimethyl-4,8-dihydrobenzo[1,4]thiazine-3,5-dione	Fruits	[41]
	148	3’,4’-dedisulphated-atractyloside	Fruits	[46]
	149	2-methyl-3-buten-2-ol-β-d-ap-iofuranosyl-(1→6)-β-d-glucopyranoside	Fruits	[51]
	150	everlastoside C	Fruits	[51]
Flavonoids	151	ononin	Fruits	[43]
	152	quercetin	Fruits	[37]
	153	allopatuletin	Fruits	[37]
	154	patuletin-3-glucuronide	Fruits	[34]
Flavonoids	155	quercetin-3-*O*-glucuronide	Fruits	[34]
	156	formononetin	Fruits	[43]
Tihiazdes	157	xanthiazone	Fruits	[36]
	158	2-hydroxy-xanthiazone	Fruits	[42]
	159	7-hydroxymethyl-8,8-dimethyl-4,8-dihydrobenzol[1,4]thiazine-3,5-dione-11-*O*-β-d-glucopyranoside	Fruits	[43]
	160	2-hydroxy-7-hydroxymethyl-8,8-dimethyl-4,8-dihydrobenzol[1,4]thiazine-3,5-dione-11-*O*-β-d-glucopyranoside	Fruits	[43]
	161	7-Hydroxymethyl-8,8-dimethyl-4,8-dihydrobenzol[1,4]thiazine-3,5-dione-(2-*O*-caffeoyl)-β-d-glucopyranoside	Fruits	[52]
Anthraquinones & naphthoquinones	162	xanthialdehyde	Fruits	[53]
	163	chrysophanic acid	Fruits	[54]
	164	emodin	Fruits	[54]
	165	aloe emodin	Fruits	[54]
	166	5-hydroxy-3,6-dimethoxy-7-methyl-1,4-naphthalenedione	Roots	[28]
Other compounds	167	5-methyluracil	Roots	[39]
	168	uracil	Roots	[39]
	169	sibiricumthionol	Fruits	[19]
	170	indole-3-carbaldehyde	Fruits	[45]
	171	N-(1’-d-deoxyxylitolyl)-6,7-dimethyl-1,4-dihydro-2,3-quinoxalinedione	Fruits	[38]
	172	nonadecanoic acid	Roots	[39]
	173	hexadecanoic acid	Leaves	[32]

**Table 3:** Pharmacological effects of *X. strumarium.*

Effects	Detail	Extracts/Compounds	Concentration/Dose	In Vivo/In vitro	Reference
***Anti-AR effects***	Inhibiting C 48/80-induced systemic anaphylaxis	WEX	Mice, 0.01–1 g/kg (p.o.)	in vivo	[61,62]
	Inhibiting histamine and TNF-α released from RPMC	WEX	RPMC, 0.01–1 mg/mL	in vitro	[63]
	Modulating the HMC-1- and PBMNC-mediated inflammatory and immunological reactions	WEX	HMC-1, PBMNC, 0.25–1 mg/mL	in vitro	[63]
	Inhibiting histamine and cAMP released from RPMC	MEX	RPMC, 20–500 μg/mL	in vitro	[64]
	Ameliorate the nasal symptoms of OVA induced AR rats via anti-allergic; down-regulating IgE; anti-inflammatory and analgesic properties	CXT	Rats, 5, 10, 20 mg/kg (p.o.)	in vivo	[65]
***Anti-tumor effects***	***Lung cancer***				
	Growth inhibition by suppression of STAT3, GSK3β and β-catenin	xanthatin	Cell lines of A549, H1975, H1299, H1650 & HCC827, 1–40 μM	in vitro	[66,67,68]
	Triggering Chk1-mediated DNA damage and destabilization of Cdc25C via lysosomal degradation	xanthatin			
	Cytotoxic effects on A549 cell	8-*epi*-xanthatin	IC_50_ = 1.1 μg/mL	in vitro	[17]
		8-*epi*-xanthatin epoxide	IC_50_ = 3.0 μM	in vitro	[69]
		xanthatin	IC_50_ = 1.3 μg/mL	in vitro	[17]
		8-epi-xanthatin-1α, 5α-epoxide	IC_50_ = 9.5 μM	in vitro	[25]
		1β-hydroxyl-5α-chloro-8-epi-xanthatin	IC_50_ = 20.7 μM	in vitro	[25]
		EEXA	IC_50_ = 52.2 μg/mL	in vitro	[70]
	***Breast cancer***				
	Cytotoxic effects on MDA-MB-231 cells	xanthatin	IC_50_ = 13.9 μg/mL	in vitro	[71]
	Cytotoxic effects on MDA-MB-231 cells	xanthinosin	IC_50_ = 4.8 μg/mL	in vitro	[71]
	Inhibiting cell growth via inducing caspase independent cell death	xanthatin	MDA-MB-231 cells, 5–25 μM	in vitro	[72]
***Anti-tumor effects***	Up-regulating GADD45 γ tumor suppressor gene; inducing the prolonged expression of c-Fos via N-acetyl-l-cysteine-sensitive mechanism	xanthatin	MDA-MB-231 cells, 2.5–10 μM	in vitro	[73,74]
	Cytotoxic effects on MFC7 cells	EEXA	IC_50_ = 70.6 μg/mL	in vitro	[70]
	***Cervical cancer***				
	Altering the antioxidant levels	WEX	Hela cells, 12.5–50 μg/mL	in vitro	[75]
	Promoting apoptosis via inhibiting thioredoxin reductase and eliciting oxidative stress	xanthatin	Hela cells, 5–20 µM	in vitro	[76]
	***Colon cancer***				
	Cytotoxic effects on HCT-15 cells	xanthatin	ED_50_ = 1.1 μg/mL	in vitro	[17]
		8-*epi*-xanthatin	ED_50_ = 0.1 μg/mL	in vitro	[17]
	Cytotoxic effects on WiDr cells	xanthatin	IC_50_ = 6.15 μg/mL	in vitro	[71]
		xanthinosin	IC_50_ = 2.65 μg/mL	in vitro	[71]
	Cytotoxic effects on BGC-823 cells	eremophil-1(10),11(13)-dien-12,8*β*-olide	IC_50_ = 13.22 µM	in vitro	[77]
		8-*epi*-xanthatin-1*β*,5*β*-epoxide	IC_50_ = 2.43 µM	in vitro	[77]
		tomentosin	IC_50_ = 4.54 µM	in vitro	[77]
	Cytotoxic effects on KE-97 cells	eremophil-1(10),11(13)-dien-12,8*β*-olide	IC_50_ = 4.41 µM	in vitro	[77]
		8-*epi*-xanthatin-1*β*,5*β*-epoxide	IC_50_ = 1.44 µM	in vitro	[77]
		tomentosin	IC_50_ = 3.47 µM	in vitro	[77]
	Inducing G2/M cell cycle arrest and apoptosis	xanthatin	MKN-45 Cells, 3.9–18.6 µM	in vitro	[75]
	Potentiating both extrinsic and intrinsic TRAIL-mediated apoptosis pathways and also decreased the level of cell survival protein Bcl-2	xanthinosin	AGS cells, 8 µM	in vitro	[18]
		lasidiol *p*-methoxybenzoate	AGS cells, 16 µM	in vitro	[18]
	Cytotoxic effects on CT26 cells	EEXA	IC_50_ = 58.9 μg/mL	in vitro	[70]
		CFEEXA	IC_50_ = 25.3 μg/mL	in vitro	[70]
	Cytotoxic effects on AGS cells	fructusnoid C	IC_50_ = 7.6 µM	in vitro	[79]
	***Liver cancer***				
	Cytotoxic effects on SNU387 cells	1*β*-hydroxyl-5α-chloro-8-*epi*-xanthatin	IC_50_ =5.1 µM	in vitro	[25]
	Cytotoxic effects on HepG2 cells	MEX	LC_50_ = 112.9 μg/mL	in vitro	[80]
		EAFMEX	LC_50_ = 68.739 μg/mL	in vitro	[80]
	Induction of apoptosis via inhibiting thioredoxin reductase and eliciting oxidative stress	xanthatin	HepG2 cells, 5–40 μM	in vitro	[76]
	***Meningioma***				
	Cytotoxic effects on SK-MEL-2 cells	xanthatin	ED_50_ = 0.5 μg/mL	in vitro	[17]
		8-*epi*-xanthatin	ED_50_ = 0.2 μg/mL	in vitro	[17]
	Inhibiting melanin synthesis through downregulation of tyrosinase via GSK3*β* phosphorylation	EEXS	Mel-Ab cells, 1–50 µg/mL	in vitro	[81]
	Inhibiting cell proliferation associated with activation of Wnt/*β*-catenin pathway and inhibition of angiogenesis	xanthatin	B16-F10 cells, 2.5–40μM	in vitro	[82]
			Mice, 0.1–0.4 mg/10 g(i.p.)	in vivo	[82]
***Anti-tumor effects***	***Leukemia***				
	Cytotoxic effects on P-388 cells	DFEEXA	IC_50_ = 1.64 μg/mL	in vitro	[83]
	Cytotoxic effects on HL-60 cells	xanthatin	IC_50_ = 52.50 µg/mL	in vitro	[84]
	Cytotoxic effects on Jurkat cells	MEX	LC_50_ = 50.18 µg/mL	in vitro	[80]
		EAFMEX	LC_50_ = 48.73 µg/mL	in vitro	[80]
	***Other tumors***				
	Cytotoxic effects on XF-498 cells	xanthatin	ED_50_ = 1.7 μg/mL	in vitro	[17]
		8-*epi*-xanthatin	ED_50_ = 1.3 μg/mL	in vitro	[17]
	Cytotoxic effects on S180 cells	WEX	Mice, 5–20 g/kg	in vivo	[85]
	Cytotoxic effects on HEP-2 cells	CEXR	12.5–100 µg/mL	in vitro	[86]
		MEXR	12.5–100 µg/mL	in vitro	[86]
***Anti-inflammatory and analgesic effects***	***Anti-inflammatory***				
	Inhibitting LPS-stimulated inflammatory	WEX	10, 100 and 1000 µg/mL	in vitro	[87]
	Inhibitting LPS-stimulated inflammatory	MEX	30, 60 and 90 mg/mL	in vitro	[88]
		xanthatin and xanthinosin	IC_50_ = 0.47 and 11.2 μM	in vitro	[89]
		MEXL	IC_50_ = 87 μg/mL	in vitro	[90]
		MEXR	50–400 μg/mL	in vitro	[91]
		WEX	0.5, 1 and 2 mg/mL	in vitro	[92]
		MEX	0–300 μg/mL	in vitro	[93]
		MEXA	0–300 μg/mL	in vitro	[94]
		xanthiumnolic E	IC_50_ = 8.73 μM.	in vitro	[26]
	Inhibiting carrageenan induced hind paw edema	MEX	100, 200 mg/kg/d (p.o.)	in vivo	[88]
		WEX	0.1, 0.5 and 1.0 g/kg, (p.o.)	in vitro	[95]
		MEXL	100, 200 and 400 mg/kg body weight.	in vivo	[90]
	Inhibiting croton-oil-induced ear edema	NFEEX	Mice, 0.5, 0.75 and 1.0 mg/ear	in vivo	[96]
	Inhibiting both PGE 2 synthesis and 5-lipoxygenase activity	xanthatin	100 and 97 mg/mL, respectively	in vitro	[84]
	Inhibiting production of TARC/CCL17 and MDC/CCL22 induced by TNF-α/IFN-γ	EEX	10 μg/mL	in vitro	[97]
	***Analgesic effect***				
	Ameliorating HCl/EtOH-induced gastritis lesions	MEXA	50 and 200 mg/kg (p.o.)	in vivo	[94]
	Analgesic effect on acetic acid-induced abdominal constriction test and a hot plate test	MEX	100, 200 mg/kg/d (p.o.)	in vivo	[88]
	Reducing the number of writhings induced by acetic acid	NFEEX	Mice, 100,200 and 400 mg/kg body wt.	in vivo	[96]
	Analgesic effect on writhing and formalin tests	WXF	0.1, 0.5 and 1.0 g/kg, (p.o.)	in vivo	[95]
	Analgesic effect on hot plate test, acetic acid induced writhing test and formalin test	EEX	250 and 500 mg/kg body weight	in vivo	[98]
***Insecticide and antiparasitic effects***	Antiplasmodial activity against *T. evansi*	EEXL	5, 50, 500 and 1000 µg/mL	in vitro	[99]
			100, 300 and 1000 mg/kg (i.p.)	in vivo	[99]
	Insecticidal effects against *T. b. brucei*	xanthatin	IC_50_ = 2.63 µg/mL	in vitro	[84]
	Anti-insect effects towards *P. viteana*	MEX	LC_50_ = 11.02 (w/w)	in vitro	[100]
***Insecticide and antiparasitic effects***	Antiplasmodial activity against *P. berghei*	EEXL	IC_50_ = 4 µg/mL	in vitro	[101]
	Insecticidal properties against C. chinensis	WEXL	1%, 2% and 4% concentration	in vitro	[102]
	Anti-nematode activity against *Meloidogyne javanica*	EEX	3%, 6% and 12% concentration	in vitro	[103]
	Insecticidal effects against *A. caspius*, *C. pipiens*	MEX	LC_50_ = 531.07 and 502.32 μg/mL, respectively	in vitro	[80]
***Antioxidant effects***	Scavenging DPPH	CEXR and MEXR	LC_50_ = 10.28 and 40.40 µg/mL	in vitro	[86]
		WEX	0.05–0.2 mg/mL	in vitro	[95]
		EEXR and CEXR	IC_50_ = 29.81 and 24.85 µg/mL	in vitro	[106]
		EEXL	IC_50_ = 85 µg/mL	in vitro	[107]
	Scavenging DPPH	hexadecanoic acid; α- amyrin; 14-methyl-12, 13-dehydro-sitosterol-heptadeconate	IC_50_ = 106.4, 64.16 and 76.18 µg/mL	in vitro	[32]
	Scavenging DPPH	EOX	138.87 μg/mL	in vitro	[108]
		MEX	Not mentioned	in vitro	[28]
	Scavenging nitric oxide	EEXR and CEXR	IC_50_ = 395.20 and 415.80 µg/mL	in vitro	[106]
		EEXL	IC_50_ = 72 µg/mL	in vitro	[107]
	Scavenging hydrogen peroxide	EEXR and CEXR	IC_50_ = 10.18 and 9.23 µg/mL	in vitro	[106]
		EEXL	IC_50_ = 62 µg/mL	in vitro	[107]
	Increasing of superoxide dismutase, glutathione peroxidase, glutathione reductase and catalase contents	PEEXW	250 and 500 mg/kg body weight (p.o for 20 days)	in vivo	[104]
	Liposome protection	WEX	0.05–0.2 mg/mL	in vitro	[95]
	Scavenging ABTS	WEX	0.05–0.2 mg/mL	in vitro	[95]
	Reducing activity	WEX	0.05–0.2 mg/mL	in vitro	[95]
	Increasing of SOD, CAT, GSH and GPx contents	MEXS	100 and 200 mg/kg (p.o., for 10 days)	in vivo	[105]
	Superoxide anion	EEXR and CEXR	IC_50_ = 495.30 and 418.30 µg/mL	in vitro	[106]
	Scavenging hydroxyl radicals	hexadecanoic acid; α- amyrin; 14-methyl-12, 13-dehydro-sitosterol-heptadeconate	IC_50_ = 127.4, 83.96 and 84.4 µg/mL	in vitro	[32]
	FRAP antioxidant activity	MEX	Not mentioned	in vitro	[28]
***Antibacterial and antifungal effects***	***Antibacterial***				
	Inhibitory effects against *V. cholerae*	WEXFT	Not mentioned	in vitro	[109]
	Inhibitory effects against *S. epidermidis, B. cereus, K. pneumoniae, P. aeruginosa and S. fyphi*	xanthatin	MIC = 31.3, 62.5, 31.3, 125 and 125 µg/mL	in vitro	[110]
	Inhibitory effects against *K. pneumoniae, P. vulgaris, P. Aeruginosa, P. putida, S. typhimurium, B. cereus, B. subtilis, S. epidermidis*	MEXL	500 and 100 mg/mL	in vitro	[111]
	Inhibitory effects against *E. coli*	β-sitosterol and β-daucosterol	MIC = 0.17 and 0.35 µg/mL	in vitro	[112]
	Inhibitory effects towards *K. pneumonia, P. mirabilis, E. coli, B. subtilis, E. faecalis, S. aureus*	MEXL	50, 100, 150, 200 and 250 mg/mL, respectively	in vitro	[113]
		WEXL			
***Antibacterial and antifungal effects***	Inhibitory effects against *S. aureus, B. subtilis, K. pneumoniae and P. aeruginosa*	EOXL	MIC = 0.5, 1.3, 4.8 and 20.5 µg/mL, respectively	in vitro	[114]
	Inhibitory effects against Shiga toxin-producing *E. coli*	EOXL	30, 60 and 120 mg/mL	in vitro	[115]
	Inhibitory effects against *S. aureus and E. coli*	WEX	MIC = 31.25 and 7.81 mg/mL, respectively	in vitro	[116]
	Inhibitory effects against *R. toxicus, S. aureus and P. S. syringae*	EOX	MIC = 25, 50 and 50 µg/mL, respectively	in vitro	[108]
	***Antifungal***				
	Inhibitory effects against *P. drechsleri*	deacetylxanthumin	MIC = 12.5 µg/mL	in vitro	[117]
	Inhibitory effects against *P. infestans*	MEX	MIC = 2.0% w/v	in vitro	[118]
	Inhibitory effects against *C. albicans and A. niger*	EOXL	MIC = 55.2 and 34.3 µg/mL, respectively	in vitro	[114]
	Inhibitory effects against *P. oryzae and F. oxysporum*	EOX	MIC = 12.5 and 50 µg/mL, respectively	in vitro	[108]
	Inhibitory effects against *A. niger, A. flavus, F. oxysporum, F. solani, A. alternata* and *P. digitatum*	EOXL	MIC = 8 µg/mL and MFC = 8 µg/mL	in vitro	[119]
***Antidiabetic effects***	Exhibiting potent hypoglycemic activity	WEX	15 and 30 mg/kg (i.p.)	in vivo	[120]
	Decreasing the plasma glucose in diabetic rats	caffeic acid	0.5–3 mg/kg (i.v.)	in vivo	[121]
	Decreasing the blood glucose and HbA1C level and increase the level of insulin	MEXS	100 and 200 mg/kg (p.o., for 30 days)	in vivo	[105]
	Inhibitory effect against rAR and rhAR	methyl-3,5-di-O-caffeoylquinate	IC_50_ = 0.30 and 0.67 µM, respectively	in vivo	[47]
	Inhibitory effect against α-glucosidase	CFMEXL	IC_50_ = 72 µg/mL	in vitro	[122]
	Inhibitory effect against α-glucosidase	MEX	IC_50_ = 15.25 µg/mL	in vivo	[28]
***Antilipidemic effects***	Decreasing plasma cholesterol, triglyceride, LDL, and VLDL and increasing plasma HDL levels	CEXR and EEXR	200 and 400 mg/kg (p.o.)	in vivo	[106]
	Improving lipid homeostasis	WEX	570 and 1140 mg/kg (p.o., for 6 weeks)	in vivo	[123]
	Decreasing blood glucose, TC, TG, LDLC levels and increasing HDLC levels.	WEX	3.7 and 11.11 g/kg (p.o., for 4 weeks)	in vivo	[124]
***Antiviral activity***	Antiviral activity against duck hepatitis B virus	WEX	0.01, 0.1 and 1 g/kg (i.g., for 10 days)	in vivo	[125]
	Antiviral activity against Influenza A virus	norxanthantolide F	IC_50_ = 6.4 µM	in vitro	[13]
		2-desoxy-6-epi-parthemollin	IC_50_ = 8.6 µM	in vitro	[13]
		xanthatin	IC_50_ = 8.4 µM	in vitro	[13]
		threo-guaiacylglycerol-8′-vanillic acid ether	IC_50_ = 8.4 µM	in vitro	[13]
		caffeic acid ethyl ester	IC_50_ = 3.7 µM	in vitro	[13]
***Other pharmacological effects***	Anti-septic activity	CXT	10, 20 and 40 mg/kg(i.p.)	in vivo	[126]
	Attenuating hepatic steatosis	WEX	570 and 1140 mg/kg (p.o., for 6 weeks)	in vivo	[127]
	Anti-arthritic effect	EEX	75 and 300 mg/kg (p.o.)	in vivo	[128]
***Other pharmacological effects***	Anti-pyretic activity	MEXW	200 and 400 mg/kg (p.o.)	in vivo	[129]
	Anti-epileptic activity	PEEXW	250 and 500 mg/kg (p.o., for 20 days)	in vivo	[130]
	Antiurolithiatic effect	HEEXB	500 mg/kg (p.o.)	in vivo	[131]
	Antiulcer effect	EEXL	200 and 400 mg/kg	in vivo	[132]
	Cardioprotective effect	CXT	10, 20 and 40 mg/kg (p.o.)	in vivo	[133]

**Table 4:** Toxicities and side effects of *X. strumarium.*

Extracts/Compounds	Animal/Subjects	LD_50_/Toxic Dose Range	Toxic Reactions	Reference
WEX	mice	LD_50_ = 201.14 g/kg (i.g., crude herb mass equivalent)	Death	[139]
WEX	mice	LD_50_ = 167.60 g/kg (i.g., crude herb mass equivalent)	Death	[140]
EEX	mice	LD_50_ = 275.41 g/kg (i.g., crude herb mass equivalent)	Death	[140]
WEX	mice	LD_50_ = 194.15g/kg (i.g., crude herb mass equivalent)	Death	[141]
carboxyatractyloside	swine	10–100 mg (i.v.)	Death	[142]
atractyloside	mice	50–200 mg/kg (i.p.)	Increasing contents of ALT, AST, ALP, MDA in mice serum	[143]
carbxyatractyloside	mice	50–150 mg/kg (i.p.)	Increasing contents of ALT, AST, ALP, MDA in mice serum	[143]
NFEEX	mice	0.06, 0.3, 0.7 g/kg (i.g., for 28 days)	Weight loss, enlarged hepatic cell space, karyolysis and inflammatory cell infiltration	[145]
WFEEX	mice	0.06, 0.3, 0.7 g/kg (i.g., for 28 days)	Weight loss, enlarged hepatic cell space, karyolysis, and inflammatory cell infiltration	[145]
WEX	mice	21.0 g/kg (i.g., for 28 days)	Weight loss and increase of ALT, AST in mice serum	[146]
WEX	mice	7.5, 15.0 and 30.0 g/kg (i.g., for 5 days)	Increasing contents of VLDL/LDL, β-HB, glutamate, choline, acetate, glucose in serum	[147]
WEX	mice	16.7 g/kg (i.g., for 7 days)	Increasing contents of GLDH, α-GST and decreasing miRNA-122	[148]
MEXA	mice	100, 200, 300 mg/kg	Depressing the action of central nervous system	[149]
atractyloside	rat hepatocytes	0.01–0.05 g/L	Reducing cell viability and intracellular GSH content	[150]
atractyloside, carbxyatractyloside	L-02 cells, BRL cells	100 μmol/L for 48 h	Inhibiting cell proliferation, improving LDH activity	[147]
WEX	HK-2 cells	100 μg/mL	Inhibiting cell proliferation	[151]
HEEXA	CHO cells	25–100 μg/mL	Inducing DNA damage	[152]
EFEEX	MIHA cells	IC_50_ = 231.1 μg/ml	Decreasing viability of cell	[153]
WEX	zebrafish	15 μg/mL	Decreasing hatch rate	[154]

## Abbreviations

WEX	water extracts of fruit of *Xanthium strumarium*
MEX	methanol extracts of fruits of *X. strumarium*
EEXA	ethanol extracts of aerial parts of *X. strumarium*
EEXS	ethanol extracts of stems of *X. strumarium*
WFEEX	water fraction of ethanol extracts of fruits of *X. strumarium*
NFEEX	n-butanol fraction of ethanol extracts of fruits of *X. strumarium*
MEXA	methanol extracts of aerial parts of *X. strumarium*
HEXA	hydroalcoholic extracts of aerial parts of *X. strumarium*
EAFEEX	ethylacetate fraction of ethanol extracts of fruits of *X. strumarium*
CFEEXA	chloroform fraction of ethanol extracts of aerial parts of *X. strumarium*
CEXR	chloroform extracts of roots of *X. strumarium*
MEXR	methanol extracts of roots of *X. strumarium*
EAFMEX	ethylacetate fraction of methanol extracts of fruits of *X. strumarium*
DFEEXA	dichloromethane fraction of ethanol extracts of aerial parts of *X. strumarium*
EEX	ethanol extracts of fruits of *X. strumarium*
MEXL	methanol extracts of leaves of *X. strumarium*
WEXL	water extracts of leaveas of *X. strumarium*
EEXL	ethanol extracts of leaves of *X. strumarium*
EEXL	ethanol extracts of leaves of *X. strumarium*
PEEXW	petroleum ether extracts of whole plant of *X. strumarium*
MEXS	methanol extracts of stems of *X. strumarium*
EEXR	ethanol extracts of roots of *X. strumarium*
EOX	essential oil of fruits of Xanthium strumarium
EOXL	essential oil of leaves of Xanthium strumarium
WEXFT	water extract of flowering twigs of Xanthium strumarium
CFMEXL	chloroform fraction of methanol extracts of leaves of *X. strumarium*
MEXW	methanol extracts of whole plant of *X. strumarium*
HEEXB	hydro-ethanol extracts of burs of *X. strumarium*
HEEXA	hydro-ethanol extracts of aerial parts of *X. strumarium*
EFEEX	ethylacetate fraction of ethanol extracts of *X. strumarium*
